# Microbiome structure variation and soybean’s defense responses during flooding stress and elevated CO_2_

**DOI:** 10.3389/fpls.2023.1295674

**Published:** 2024-02-08

**Authors:** Lauryn Coffman, Hector D. Mejia, Yelinska Alicea, Raneem Mustafa, Waqar Ahmad, Kerri Crawford, Abdul Latif Khan

**Affiliations:** ^1^ Department of Engineering Technology, Cullen College of Engineering, University of Houston, Sugar Land, TX, United States; ^2^ Department of Biological Sciences and Chemistry, College of Natural Science and Mathematics, University of Houston, Houston, TX, United States

**Keywords:** microbiome, diversity, flooding stress, climatic CO2, gene expression, oxidative stress, soybean

## Abstract

**Introduction:**

With current trends in global climate change, both flooding episodes and higher levels of CO_2_ have been key factors to impact plant growth and stress tolerance. Very little is known about how both factors can influence the microbiome diversity and function, especially in tolerant soybean cultivars. This work aims to (i) elucidate the impact of flooding stress and increased levels of CO_2_ on the plant defenses and (ii) understand the microbiome diversity during flooding stress and elevated CO_2_ (eCO_2_).

**Methods:**

We used next-generation sequencing and bioinformatic methods to show the impact of natural flooding and eCO_2_ on the microbiome architecture of soybean plants' below- (soil) and above-ground organs (root and shoot). We used high throughput rhizospheric extra-cellular enzymes and molecular analysis of plant defense-related genes to understand microbial diversity in plant responses during eCO_2_ and flooding.

**Results:**

Results revealed that bacterial and fungal diversity was substantially higher in combined flooding and eCO_2_ treatments than in non-flooding control. Microbial diversity was soil>root>shoot in response to flooding and eCO_2_. We found that sole treatment of eCO_2_ and flooding had significant abundances of *Chitinophaga, Clostridium*, and *Bacillus*. Whereas the combination of flooding and eCO2 conditions showed a significant abundance of *Trichoderma* and *Gibberella*. Rhizospheric extra-cellular enzyme activities were significantly higher in eCO_2_ than flooding or its combination with eCO_2_. Plant defense responses were significantly regulated by the oxidative stress enzyme activities and gene expression of *Elongation factor 1* and *Alcohol dehydrogenase 2* in floodings and eCO_2_ treatments in soybean plant root or shoot parts.

**Conclusion:**

This work suggests that climatic-induced changes in eCO_2_ and submergence can reshape microbiome structure and host defenses, essential in plant breeding and developing stress-tolerant crops. This work can help in identifying core-microbiome species that are unique to flooding stress environments and increasing eCO_2_.

## Graphical Abstract

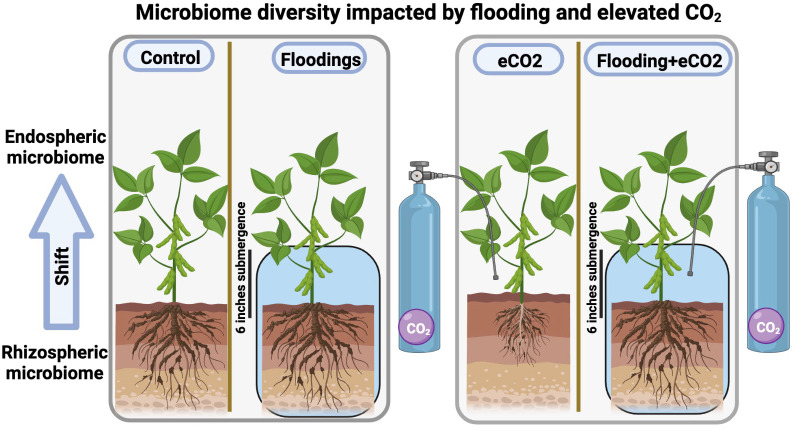

## Introduction

Climate change decreases plant productivity and threatens food security (Ahmad and Prasad, 2011). Climate changes are interconnected and multifaceted. Greenhouse gas emissions, specifically CO_2_, are increasing, leading to changes in global temperature and rainfall patterns. The IPCC reported that with global warming of 1.5°C, there will be more flooding in coastal and low-lying cities and local areas experiencing increased frequency and intensity of rain. In 2019 alone, flooding along three major rivers caused roughly $20.3B in damage, affecting agriculture and infrastructure [NOAA National Centers for Environmental Information (NCEI), 2018]. The increased amount of water available or excess submergence is hazardous to plant growth and productivity.

Flooding broadly comes in two forms: waterlogging, where water is on the soil surface and only plant roots are surrounded by water. The other form is called submergence, where the whole plant can either be underwater/fully submerged or partially submerged (Jia et al., 2021). Hypoxia is caused in both cases by a lack of oxygen in the plants (Loreti and Perata, 2020). Submergence, studied here, causes excessive hypoxia (Lee et al., 2011). It exacerbates subsidiary stresses such as pathogenesis, herbivory (Hsu and Shih, 2013), and soil nutrient balance (Hurkman, 1992; Degenhardt et al., 2000; Zhu, 2001; Yang et al., 2008; Valliyodan et al., 2016). Hypoxia induces the production of reactive oxygen species (ROS; superoxide O_2_^–^, singlet oxygen _1_O^2^, hydrogen peroxide H_2_O_2_) that damage the functional proteins, lipids, carbohydrates, and nucleic acid in plants (Boyarshinov and Asafova, 2011; Boogar et al., 2014). While other factors, such as soil nutrient availability, can influence soil microbiome during flooding, the overwhelming factor is the lack of oxygen (Unger et al., 2009). A study has shown that soil type, soil moisture, and field slope can influence bacterial movement in flooded soils, but this would be specific (Callahan et al., 2017b) and is outside of the scope of this study.

Crop plant flooding events are estimated to decrease yields by 50%–80% (Mittler and Blumwald, 2010; Nanjo et al., 2014; Cooke and Leishman, 2016; Sasidharan et al., 2017). Flooding’s impact on the agriculture economy costs more than $5.5 billion in the United States, whereas climate change impacts are estimated to range up to $1.5 trillion globally. Soybeans are in the top 5 important food crops around the world (Savary et al., 2019), which is mostly due to their essential amino acid composition and complete protein content (Michelfelder, 2009). There have been many studies investigating the physiological and/or biochemical effects of flooding on soybeans (Khan et al., 2021; Komatsu et al., 2021; Staniak et al., 2023; Wang and Komatsu, 2018; Zhou et al., 2021), but few studies have investigated the shifts of its microbial communities (Lian et al., 2023; Yu et al., 2022). For example, it has been shown that flooding stress creates signaling for cell death and proteolysis in the root tips (Yanagawa and Komatsu, 2012; Nanjo et al., 2013), along with diminished root elongation and hypocotyl pigmentation (Hashiguchi et al., 2009). Soybeans and other legumes are potentially more sensitive to flooding due to lack of oxygen, having a negative impact on nitrogen fixation in the root systems (Shimamura et al., 2002; Yamauchi et al., 2013; Souza et al., 2016). However, soybeans generate aerenchyma throughout the plant, termed “secondary” aerenchyma, to cope with flooding stress (Shimamura et al., 2003).

Plant molecular response pattern to stress triggers the gene expression profile, and biosynthetic pathways enable signal transduction to produce biochemical metabolites and enzymes that increase the defense responses of plants (Ahuja et al., 2010; Godoy et al., 2021; Razi and Muneer, 2021). For example, *SnRK1* directly binds to the promoter regions of hypoxia-inducible genes in response to submergence (Park et al., 2020). In plants, the enzymes pyruvate decarboxylase (PDC) and alcohol dehydrogenase (ADH) are crucial players during low oxygen conditions (Jardine and McDowell, 2023; Strommer and Garabagi, 2009). However, more needs to be understood about how these molecular signaling events correspond to microbial symbionts also affected by climatic conditions.

CO_2_, on the other hand, is essential to plant photosynthesis; however, it can negatively impact plant growth and physiology (Gojon et al., 2023). The interaction of water and CO_2_ is well known. The elevated CO_2_ (eCO_2_) produces weak carbonic acid, which causes root cell wall acidification (Tan and Zwiazek, 2019). This impacts the root architecture and changes the rhizospheric soil chemistry, where any change in the rhizosphere can also influence microbial community structures. Furthermore, eCO_2_ mainly lowers the nitrogen content of plant tissues, possibly through specific inhibition of nitrate uptake and assimilation (Tausz-Posch et al., 2020). The altered nutrient status of plants grown at eCO_2_ is one likely cause of the acclimation of photosynthesis to eCO_2_ that prevents complete stimulation of biomass production in response to “CO_2_ fertilization” (Cotrufo et al., 1998). The high natural genetic variability of the eCO_2_ impact on plant nutrient status can be exploited as a promising strategy to breed future crops better adapted to a high-CO_2_ world (Tausz et al., 2017). eCO_2_ and flooding separately drastically impact the agricultural production system. Water has a lower gas exchange rate than air, reducing gas exchange in the soil while already in a higher CO_2_ environment, limiting oxygen availability more. Elevated CO_2_ levels have the potential to be either beneficial or detrimental. Thus, eCO_2_ and flooding-induced hypoxia can impact the plant’s ability to tolerate stress and influence the associated microbial communities, which has not been fully elucidated (Jones et al., 2018).

Microbes, conversely, improve plant growth, productivity, and resistance against pathogenicity and abiotic stresses (D’hondt et al., 2021; Lyu et al., 2021). Recently, the plant-associated microbiome has been coined as a “second genome” highly variable in diversity, abundance, and composition (Pfeiffer et al., 2017). Some recent studies have explained the role of the microbiome in drought and heat stress conditions (Jorquera et al., 2016; Citlali et al., 2018; Delgado-Baquerizo et al., 2018; Mandakovic et al., 2018; Araya et al., 2020; Astorga-Eló et al., 2020; Khan et al., 2020b); however, how microbial communities respond to eCO_2_ and hypoxia-induced flooding has not been fully explained. Stressors such as flooding can cause a shift in a plant’s root exudates, the main mode of communication for the rhizospheric microbiome (Vives-Peris et al., 2020; Martínez-Arias et al., 2022). It is established that abiotic stress changes root exudates, influencing the microbiome (Vargas et al., 2020; Martínez-Arias et al., 2022). Developing “secondary” aerenchyma can release oxygen to aid beneficial microbes during abiotic stressors such as flooding (Bodelier, 2003). Gaining popularity recently is the phyllosphere which encompasses the aboveground portions of the plant from the leaves, stems, fruits, and flowers (Bashir et al., 2022). The phyllosphere microbiome composition can shift by host, season, pollution, and location (Bao et al., 2020; Qian et al., 2020; Sohrabi et al., 2023). Still, a knowledge gap exists on how phytomicrobiome, populations, and function can improve crop stress tolerance (Khan et al., 2015; Khan et al., 2020b; Trivedi et al., 2020). Increasing our mechanistic understanding and real-world understanding of microbiome–plant interactions under flooding stress offers enormous potential for increasing the resilience of plants in such conditions (Van Der Heijden and Hartmann, 2016; De Vries et al., 2020).

Looking at the current focus on plant–microbe interactions, there is also a significant need to harness stress tolerance mechanisms to improve plant growth in extreme conditions and focus on increasing plant yields (Hussain et al., 2018). Since the two factors—i) increased eCO_2_ and ii) floodings—are extremely important to plant life, it is expected that flooding more strongly influences the rhizosphere microbiome while eCO_2_ significantly influences the phyllosphere microbiome. Here, we hypothesize that flooding and eCO_2_ exposure can influence the microbiome diversity in the rhizosphere and phyllosphere of soybean plants. Both factors can also influence the microbial abilities to produce rhizospheric enzymes and plant stress tolerance by regulating oxidative stress and stress-related gene expressions. However, these adaptive mechanisms at the molecular, biochemical, and metabolite levels vary across different species of plants, their growth conditions, and exposure to stress factors. This work will provide new insights into how increased flooding and elevated carbon dioxide levels caused by global warming will have a novel impact on plant stress response and microbiome structure. While this study only scratches the surface of plants’ responses, it provides new questions for future studies. For this purpose, in the current study, we aim to i) elucidate the impact of flooding stress and increased eCO_2_ on the plant defenses and ii) understand the changes in microbial communities’ structure during flooding stress.

## Results

### Flooding and eCO_2_ exposure impact plant growth and oxidative stress enzymes

The results showed that the treatments impacted plant growth and development compared with control plants. Morphologically, flooding stress caused 27% fewer leaves and 38% higher internode length than the control. Overall, the sole or combined treatments of flooding and/or eCO_2_ have significantly (*p*< 0.05) hindered the plant growth (shoot and root length, biomass, number of leaves, and internode distances) compared with non-flooded control plants (**Supplementary Table 1**). A similar negative impact was also observed for the photosynthetic pigments in the combined flooding and/or eCO_2_ treatments. We found that chlorophyll contents (chl-*a* and chl-*b*) were significantly lower (*p*< 0.05) in flooding and flooding + eCO_2_ compared with control soybean plants. Combined flooding and eCO_2_ interaction was significant (*p*< 0.05). Both control and flooding showed insignificant quantities of carotenoids, whereas the eCO_2_ treatment with or without flooding was significantly lower than the rest (**Supplementary Figure 1**).

**Figure 1:**
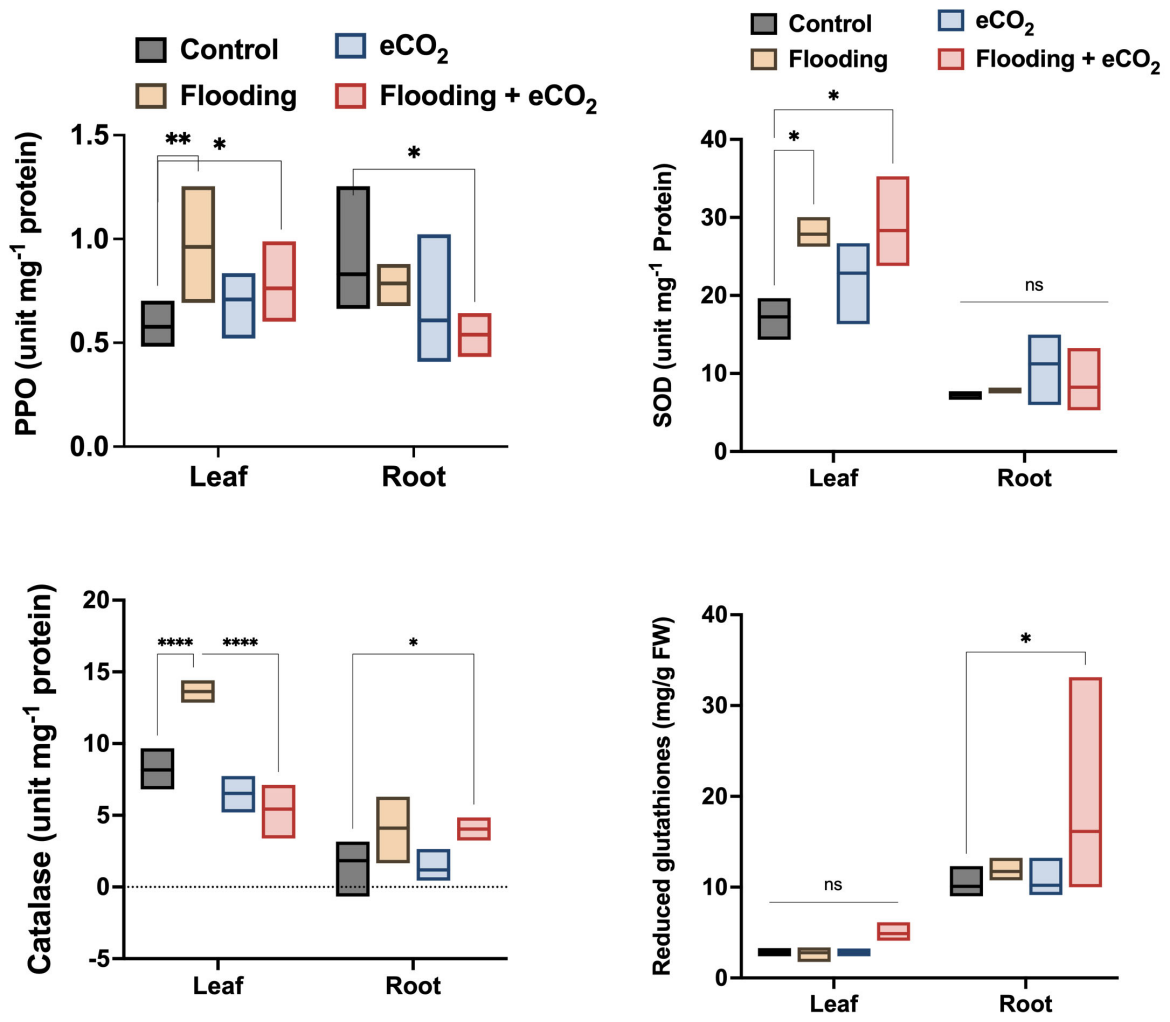
Influence of eCO_2_ and flooding on the oxidative stress-related enzymes and biochemicals. PPO, SOD, CAT, and Glut were assessed from the leaf and root parts of the soybean plants treated with eCO_2_, flooding, and eCO_2_ + flooding and compared with non-flooded control plants. The values in the bar are the mean values of three replicates and show standard deviation. The bars showing *, **, and **** are significantly different (p<0.05) in their content compared with the control as analyzed by two-way ANOVA.

The flooding stress causes significant oxidative stress, evidenced by the increased antioxidant enzyme activities. PPO (polyphenol oxidase) activities were significantly higher (*p*< 0.01; 26.2%) in the leaf part during flooding stress compared with other treatments and control soybean plants (**Figure 1**). The PPO activities were comparatively reduced in eCO_2_ and in combination with flooding stress. The peroxidase (POD) activities were non-significantly higher in the leaf during different treatments than in the control. POD activity was also non-significantly regulated in root parts across different treatments compared with the control (**Supplementary Figure 2**). However, this was still insignificant compared with the control. In the case of superoxide dismutase (SOD), it was significantly increased (*p*< 0.05; 21.4% to 29.1%) in the leaf parts of plants treated with flooding both in ambient CO_2_ and eCO_2_ applications as compared with the control.

On the contrary, the antioxidant enzyme activity in root parts was exponentially lower in all treatments (**Figure 1**). In the case of H_2_O_2_ scavenger, catalase activities were significantly higher (*p*< 0.001; 31%) in flooding stress than eCO_2_ with or without flooding stress conditions and control plants. The catalase enzyme activities were significantly lower in the root parts. However, we observed a similar trend of increased catalase activities in flooding stress conditions (**Figure 1**). Contrarily, the root parts treated with eCO_2_ with flooding stress have shown significantly (*p*< 0.05) higher catalase activities than control plants (**Figure 1**). We also assessed the contents of reduced glutathione in the root and shoot parts of different treatments. We found that reduced glutathione is significantly higher in root than leaf parts during other treatments. The root parts treated with eCO_2_ with flooding stress have shown significantly (*p*< 0.05; 18%) higher glutathione content than control plants (**Figure 1**).

### Flooding and eCO_2_ regulate microbiome diversity

Since flooding stress has significantly influenced plant growth and oxidative stress enzyme activities, we hypothesized that it would also impact the diversity and abundance of microbial communities across different treatments. For this purpose, an in-depth amplicon sequencing of 16S rRNA and ITS regions of different treatments (control, flooding, eCO_2_, and flooding + eCO_2_) was performed, followed by bioinformatics analysis. We obtained 1.93 million reads and 1.41 million reads for soil’s bacterial and fungal communities, with post-filtration of sequences assigned to chloroplast, mitochondria, and archaea. Similarly, we obtained 3.9 and 3.7 million reads from the shoot/root parts of the plants (**Supplementary Tables 2–5**). We observed 1.2 to 1.3 million bacterial amplicon sequence variants (ASVs) and 0.9 to 1.2 million fungal ASVs. ASV methods first infer biological sequences from a sample and distinguish sequence variants that differ by more than one nucleotide then analyze amplification and sequence errors (Callahan et al., 2017a). We observed that bacterial and fungal ASVs were significantly (*p*< 0.01; 28%) higher in combined flooding and eCO_2_ treatments. The bacteria and fungi ASVs were 1.3 and 1.24 million for flooding + eCO_2_. This was followed by eCO_2_ treatment which had a moderate impact on microbial ASVs (**Supplementary Table 6**).

Flooding stress showed lower ASVs than eCO_2_ treatments in fungal communities. In the different organs of the plants, the root/shoot parts of flooding + eCO_2_ showed higher (*p*< 0.01; 22%) ASV compared with control and other treatments. This was true for both bacterial and fungal ASVs. This suggests that combining flooding and eCO_2_ treatments significantly increases microbial communities’ abundances compared with control and sole flooding/eCO_2_ treatments (**Supplementary Table 6**).

**Figure 2:**
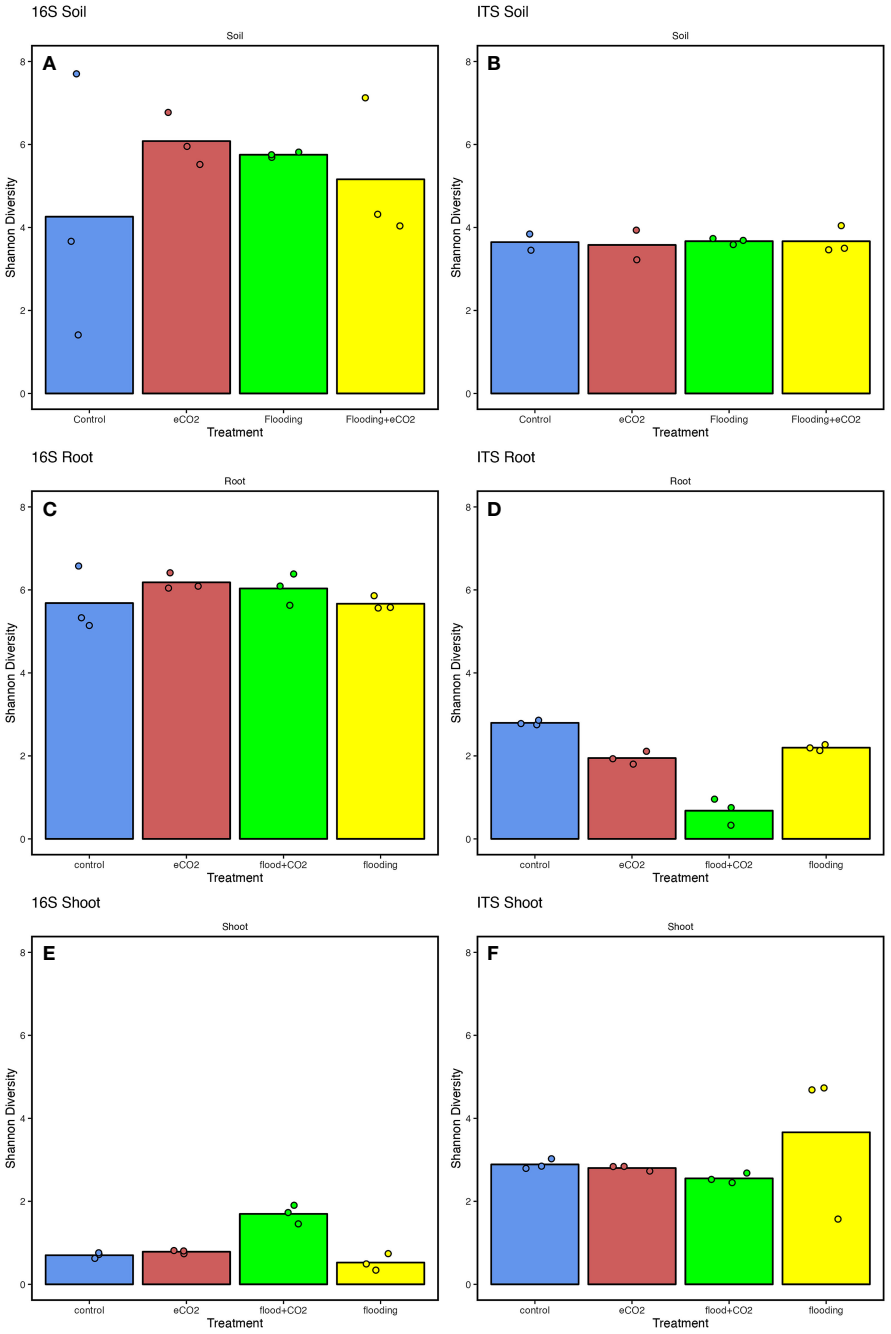
The microbiome diversity indices of soybean plants are treated with flooding stress with or without exposure to eCO_2_. The results are compared with non-flooded control soybean plants, represented in blue. Treatment with eCO_2_, flooding with eCO_2_, and flooding are represented with red, green, and yellow, respectively. **(A, B)** The bacterial (16S) and fungal (ITS) Shannon diversity indices of rhizospheric soil across treatments compared with the control. **(C, D)** The bacterial and fungal diversity of root parts of soybean plants treated with flooding stress and eCO_2_. **(E, F)** The bacterial and fungal diversity of shoot parts of soybean plants exposed to flooding and eCO_2_ compared with control plants. The data analyzed represent three replicates for each treatment (control, floodings, eCO_2_, and flooding + eCO_2_).

Overall, the results showed significantly higher (*p*< 0.05; ~6) Shannon diversity indices in the root parts than in the shoot parts (~0.5) (**Supplementary Table 7**). Among the treatments for the rhizospheric soil, the results showed significantly higher (35.2%) bacterial diversity in eCO_2_ treatments compared with the control. This was followed by flooding and flooding + eCO_2_ treatments with 29.8% and 19.1% higher bacterial diversity than control, respectively (**Figure 2**). Contrarily, the fungal diversity averaged ~3.5% for all treatments, insignificantly higher in flooding and flooding + eCO_2_ (**Figure 2**). In the endospheric microbiome, bacterial diversity was the highest in flooding and flooding + eCO_2_ treatments in the root parts (**Figure 2**). Conversely, the fungal diversity significantly reduced (121.8%) across all treatments compared with the control in roots. In the case of the shoot, a very low bacterial diversity was observed with a Shannon value of 0.70, 0.78, and 0.52 for the control, eCO_2_, and flooding + eCO_2_, respectively (**Figure 2**). Interestingly, bacterial diversity was significantly higher in flooding (1.70) compared with other treatments. Overall, flooding and eCO_2_ caused a significant (*p*< 0.05; 105.6% and 28.9%, respectively) increase in bacterial diversity compared with control, suggesting that both impact the microbiome structure. In contrast to bacterial diversity, fungal diversity in the shoot was significantly higher (*p*< 0.05; 23.6%) in flooding stress conditions compared with the control (**Figure 2**). In bacterial microbiomes, the control treatment is distributed unevenly across principal coordinates in rhizospheric soil samples compared with other treatments. Not surprisingly, samples with similar community diversity were observed for eCO_2_ and flooding + eCO_2_. The root and shoot samples were clustered adjacent throughout the microbial diversity, with replicates of flooding and flooding + eCO_2_ (**Supplementary Figure 3**).

The rhizospheric soil showed that combined factors of flooding+ eCO_2_ had significantly enriched ASVs than control vs. eCO_2_ or control vs. flooding for bacterial and fungal diversity (**Supplementary Table 8**). In the case of the root endosphere, a relatively different trend of upregulated ASV enrichment was observed in control vs. flooding than in control vs. flooding + eCO_2_ for bacterial communities. The relative fungal abundances were significantly higher in control vs. flooding + eCO_2_ than in the other treatments in the root part. A similar trend of increased bacterial ASV enrichment was observed for shoot endosphere in control vs. flooding + eCO_2_ than other treatments (**Supplementary Table 8**).

### Microbiome players in flooding and eCO_2_

#### Bacterial biomes distribution in treatments

**Figure 3:**
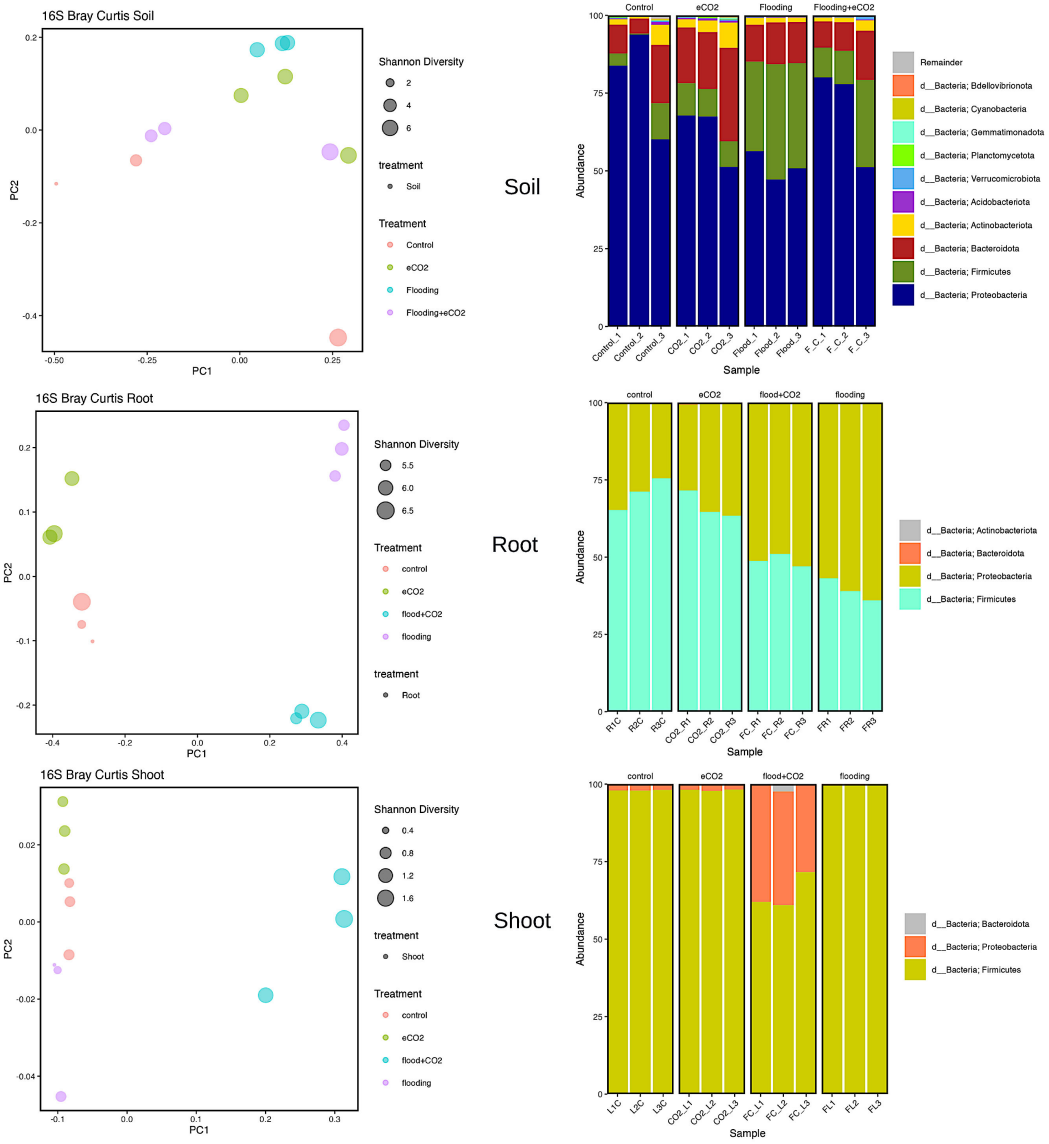
Bacterial biome diversity and phyla abundance across different treatments. The Bray–Curtis statistical analysis was used to determine bacterial microbiome variation during flooding, eCO_2_, and flooding + eCO_2_ and compared with the control. The bacterial biome of the host organ in terms of rhizosphere and endosphere was analyzed.

*Proteobacteria*, *Actinobacteria*, *Bacteroidota*, and *Firmicutes* were the significantly abundant phyla across all treatments in the rhizospheric soil. *Proteobacteria* were highly abundant (*p* > 0.05; 79%) in control, followed by 51% abundance in flooding. In *Proteobacteria*, the significant abundant families were Caulobacteraceae, Rhizobiaceae, Xanthobacteraceae, Sphingomonadaceae, Burkholderiaceae, Comamonadaceae, Pseudomonadaceae, and Rhodanovacteraceae (**Supplementary Table 9**; **Figure 3**). Of these eight families, Caulobacteraceae had 4% abundance in control and flooding and 5.5% and 7% in eCO_2_ and flooding + eCO_2_, respectively. Sphingomonadaceae was ~14% abundant across all treatments compared with the control (~8%). Pseudomonadaceae, on the other hand, had significantly higher abundances of 52%, 37%, 20%, and 18% in control, flooding + eCO_2_, flooding, and eCO_2_. Overall, the eCO_2_ treatment showed higher abundances of these families. Similarly, in the case of phyla *Bacteroidota*, the relative abundance (22%) was significantly higher in eCO_2_ compared with other treatments (11% to 13%). Chitinophagaceae family abundances were substantially lower in control (7%) compared with 10%–11% in flooding and flooding + eCO_2_ treatment. Contrarily, Chitinophagaceae was 20% abundant in eCO_2_ (**Figure 3**; **Supplementary Figure 4**). The relative abundance of *Actinobacteriota* phylum stayed relatively consistent, with a percentage between 2% and 5%. The *Actinobacteriota* comprised 21 families, and their abundances were significantly lower (>1%). The phyla *Firmicutes* was considerably higher in flooding (33%) than in control (5%). *Firmicutes* were composed of two Bacillaceae and Clostridiaceae families. The Bacillaceae was 2% abundant in eCO_2_ and control, whereas it was ~4% in flooding + eCO_2_ and flooding. However, Clostridiaceae accounts for a large abundance in flooding treatment at 26% abundance. Of the other treatments, Clostridiaceae has the lowest abundance in the control, with only 1.4% abundance, followed by eCO_2_, then flooding + eCO_2_ with 6% and 8%, respectively (**Supplementary Table 9**; **Figure 3**).

Two significantly abundant phyla (*Firmicutes* and *Proteobacteria*) were in the root and shoot. The *Firmicutes* were highly prevalent in control (71%) and eCO_2_ (66.5%). Out of the three *Firmicutes* families, the Bacillaceae was 70% abundant in control, 66% in eCO_2_, 42% in flooding + eCO_2_, and 31.5% in flooding. This was followed by many unidentified having less than 1% abundance in control and eCO_2_ but ~7% in flooding and flooding + eCO_2_. In contrast, *Proteobacteria* was the abundant phyla in both flooding + eCO_2_ and flooding, 51% and 61%, respectively. The data showed that there are nine families of *Proteobacteria*. The highly abundant families were unidentified and had an 8% abundance in eCO_2_ treatment, 4% in control, and less than 1% in flooding + eCO_2_ and flooding. The family Parvularculaceae had 3% abundance for eCO_2_ and control but less than 1% for flooding and flooding + eCO_2_. Sphingomonadaceae was also low in flooding and flooding + eCO_2_ at roughly 2% abundance compared with the 4% and 5% of control and eCO_2_, respectively. Contrarily, Alcaligenaceae and Pseudomonadaceae were significantly abundant families (42% and 15% flooding and control and 31% and 17% flooding + eCO_2_, respectively). Both control and eCO_2_ had a 13% abundance of Alcaligenaceae. eCO_2_ had only a 1% abundance of Pseudomonadaceae, while the control had approximately 4%. The shoot had a higher (65%) diversity of the phyla *Firmicutes* followed by *Proteobacteria* (34%). The relative abundance of *Firmicutes* increases to 98% flooding stress (**Supplementary Table 9**).

### Fungal biome distribution in stress

**Figure 4:**
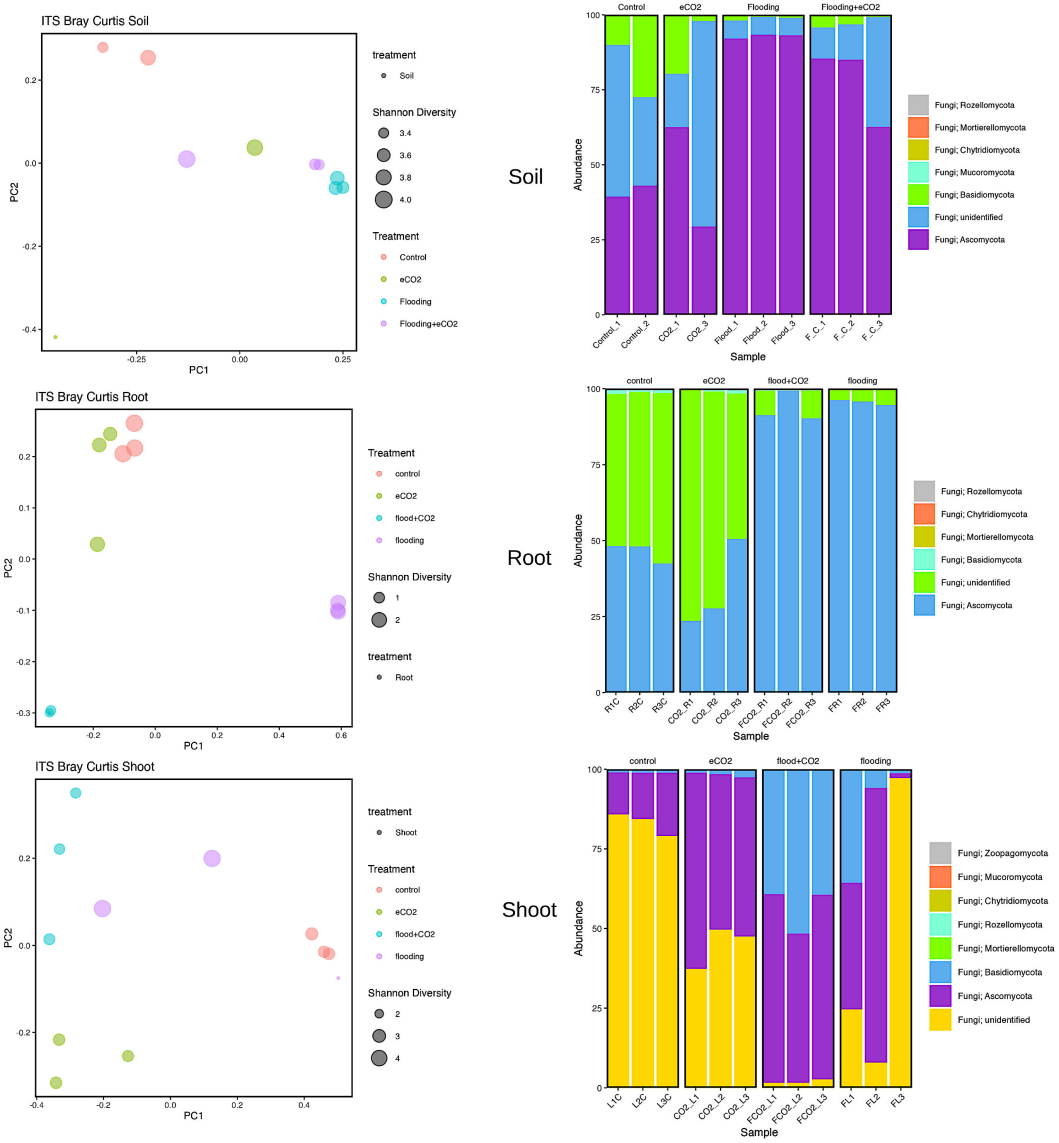
Fungal biome diversity and phyla abundance across different treatments. The Bray–Curtis statistical analysis was used to determine bacterial microbiome variation during flooding, eCO_2_, and flooding + eCO_2_ and compared with the control. The bacterial biome of the host organ in terms of rhizosphere and endosphere was analyzed.

Our results showed that two major fungal phyla (*Ascomycota* and *Basidiomycota*) were significantly abundant. Rhizospheric soil analysis showed an increase (92%) in *Ascomycota* during flooding compared with the control (41%). Flooding + eCO_2_ showed a rise in *Ascomycota* phyla to 78%. The most abundant *Ascomycota* families are Aspergillaceae, Thermoascaceae, Trichocomaceae, and Didymellaceae (**Supplementary Table 10**; **Figure 4**). The Aspergillaceaea family increases by approximately 5% in abundance during flooding with or without increased eCO_2_. The opposite is true for Thermoascaceae, which increased to approximately 43% during flooding stress and 38% with flooding + eCO_2_ compared with the control and eCO_2_ (11% and 15%, respectively). The Trichocomaceae family remained in approximately 1%–3% abundance across all treatments. We found that *Basidiomycota* was less abundant in flooding. *Basidiomycota* is 19% for control and 12% for increased eCO_2_; when flooding occurs, the abundance reduces to 3% with eCO_2_ and 1% without elevated eCO_2_ (**Figure 4**; **Supplementary Figure 5**). Rhynchogastremataceae is abundant in control and decreases with stress. The highest relative abundance was found in flooding (1%), then flooding + eCO_2_ (2%), with eCO_2_ (10%) being the least affected. There are unidentified fungal species with no assignment to phyla for approximately 42% presence in control and eCO_2_ treatments and reduced to half during flooding + eCO_2_ stress to 20% and a more significant drop in flooding of 6% (**Supplementary Table 10**).

Similarly, the root had 94% and 96% abundance of *Ascomycota* in flooding + eCO_2_ and flooding, respectively, followed by unidentified microbes. The most abundant *Ascomycota* families were Didymellaceae, Hypocreaceae, Nectriaceae, and Ophiostomatac. Didymellaceae were present in the control treatment at approximately 5% abundance and less than 1% in the eCO_2_ with and without floodings. The flooding treatment showed a 30% abundance of the family Didymellaceae. Hypocreaceae was 90% abundant in flooding + eCO_2_. The Nectriaceae family was significantly abundant (59%) during flooding, while in the flooding + eCO_2_, it was negligible. Both eCO_2_ and control had Nectriaceae at 2% and 4% lower levels, respectively. The Ophiostomatac family is in control at 4% abundance and essentially 0% in all other treatments (**Supplementary Table 10**; **Figure 4**).

In the case of shoot, the *Ascomycota* was 16% in control, 43% in flooding, 55% in flooding + eCO_2_, and 53% in abundance in eCO_2_. The prominent families in *Ascomycota* were Cladosporiaceae, Didymellaceae, Pleosporaceae, Aspergillaceae, Thermoascaceae, Trichocomaceae, Hypocreaceae, and Nectriaceae. The Cladospriaceae was abundant (18%) in flooding + eCO_2_; in sole flooding, it was 1.5% compared with control and other treatments. The Didymellaceae shows a decrease in eCO_2_ with disregard to flooding stress. There is 4% and 6% abundance during flooding and control. Pleosporaceae has 4% abundance with flooding stress but essentially zero abundance for all other treatments. Aspergillaceae and Thermoascaceae species were abundant in all treatments ranging from 1% to 5%. Trichocomaceae is present in roughly 0% abundance for control and flooding + eCO_2_ but has 1% abundance in eCO_2_ and 3.5% abundance in flooding stress. The Hypocreaceae family is abundant for all stress treatments, from 2% abundance in control to 11% in flooding, 25% in flooding + eCO_2_, and 42% in eCO_2_. Nectriaceae is more prevalent in control and flooding treatments at 2% and 4%, while eCO_2_ and flooding + eCO_2_ were absent. Finally, *Basidiomycota* has low abundance in both control and eCO_2_ with a max of 2% abundance, followed by 14% in flooding conditions and 43% in flooding+ eCO_2_ levels. The family Podoscyphaceae has a 10% abundance during flooding stress but nearly none in all other treatment conditions. The family Rhynchogastremataceae has less than 1% abundance in control, eCO_2_, and flooding stress, but when flooding + eCO_2_ are both present, it is noted that it makes up 43% of the total microbial abundance (**Supplementary Table 10**).

### Genera-level abundance across treatments

**Figure 5:**
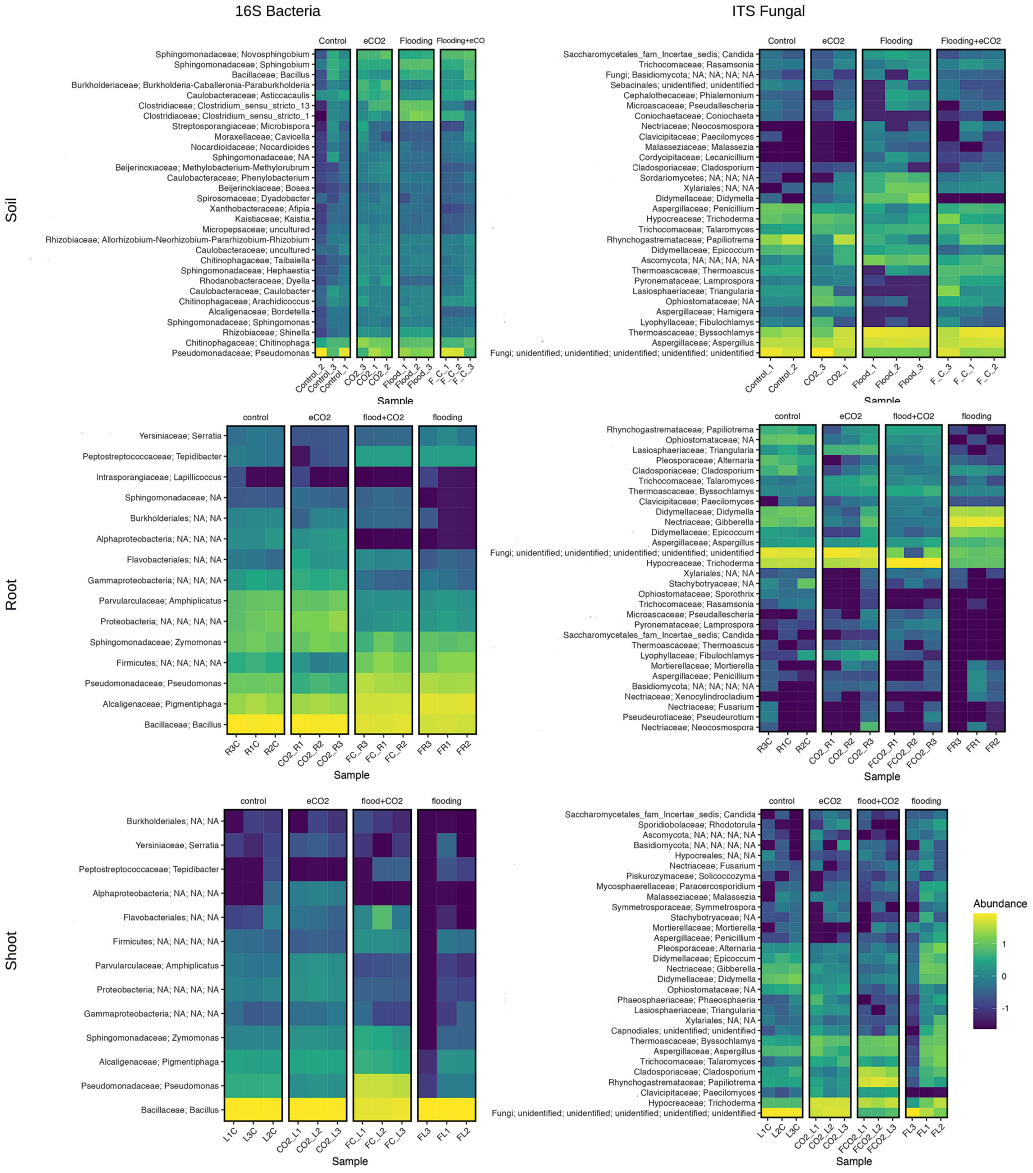
Genus-level microbiome diversity and abundance during flooding and eCO_2_ treatments. The heatmap shows the top 20 microbiome species in the rhizosphere (soil) and endosphere (root and shoot).

In the case of bacterial genera, the most abundant was *Chitinophaga*, with approximately 16% relative abundance in rhizospheric soil of eCO_2_ and between 5% and 8% abundance for other treatments. During flooding, the two highly abundant genera were *Clostridium sensu stricto 1* (14%) and *Clostridium sensu stricto 13* (10%). The main genus of the family Caulobacterales was *Asticcacaulis*, which is most abundant in eCO_2_ (**Figure 5**). Several genera of the family Sphingomonadaceae were also found. For example, *Novosphingobium* was the most abundant genus present during eCO_2_ (6.5% eCO_2_ and 7% flooding + eCO_2_). However, it was 3.5% in flooding compared with 2% relative abundance in control. The *Sphingobium* was also higher during flooding stress (7%) than that of eCO_2_ (3.5% eCO_2_ and 4% flooding + eCO_2_). The control had the lowest relative abundance of 2%. The other genera were *Burkholderia–Caballeronia–Paraburkholderia* higher in eCO_2_ at 5%, but flooding and flooding + eCO_2_ had less than 2% relative abundance. Both control and flooding treatments had less than 1% of *Burkholderia–Caballeronia–Paraburkholderia* present. The root bacterial genera *Bacillus* was the most abundant ranging from 31.5% to 70% across all treatments. The control and eCO_2_ treatments had less than 1%; for the Alcaligenaceae family, all relative abundance was represented by the genus *Pigmentiphaga*. Of the Pseudomonadaceae family, the genus of representation was *Pseudomonas*. Shoot 16S had two highly abundant genera: *Bacillus* and *Pseudomonas* (**Figure 5**).

Rhizospheric soil had two genera of the Didymellaceae family, which had high relative abundance: Didymella with 5% relative abundance in flooding and Epicoccum with 2% relative abundance in control. Aspergillaceae had three genera, one having a 0% relative abundance. The other two genera were *Penicillium*, with almost 4% relative abundance in control. *Aspergillus* had 27%–30% relative abundance during flooding regardless of eCO_2_ exposure. There were only two genera for Thermoascaceae, with *Byssochlamys* being the most abundant and *Thermoascus* having less abundance. Of the family Lasiophaeriaceae, the genus *Triangularia* was present (1%) in eCO_2_ and 3% in abundance in flooding + eCO_2_ treatments. The genus *Papilotrema* had high abundance in the control treatment at 18%, falling to 9% in the increased eCO_2_ treatment. *Papilotrema* declined in the flooding + eCO_2_ treatment to 2.5% and finally to less than 1% in the flooding treatment (**Figure 5**).

In the endosphere, the roots of two major genera from *Didymella* and *Epicocum* belong to the Didymellaceae family. Importantly, the genus *Gibberella* had 4% abundance in control, lowering to 1% in eCO_2_ treatment and 0% in flooding + eCO_2_ treatment; however, the relative abundance increased to 59% during flooding treatment. For the shoot part, the genus *Cladosporium* showed high abundance in flooding + eCO_2_ treatment at 18% and 1.5% in flooding. The abundance of *Cladosporium* for both eCO_2_ and control was less than 1%. *Trichoderma* is a highly abundant species, showing an increase of 42% in eCO_2_, 25% in flooding + eCO_2_, and 11% in flooding. *Trichoderma* only had a 2% relative abundance in the control treatment. *Papiliotrema* is a genus with a high abundance of 43% in the flooding + eCO_2_ treatment, while other treatments had less than 1% relative abundance (**Figure 5**).

### Differential abundance of taxon in treatments

**Figure 6:**
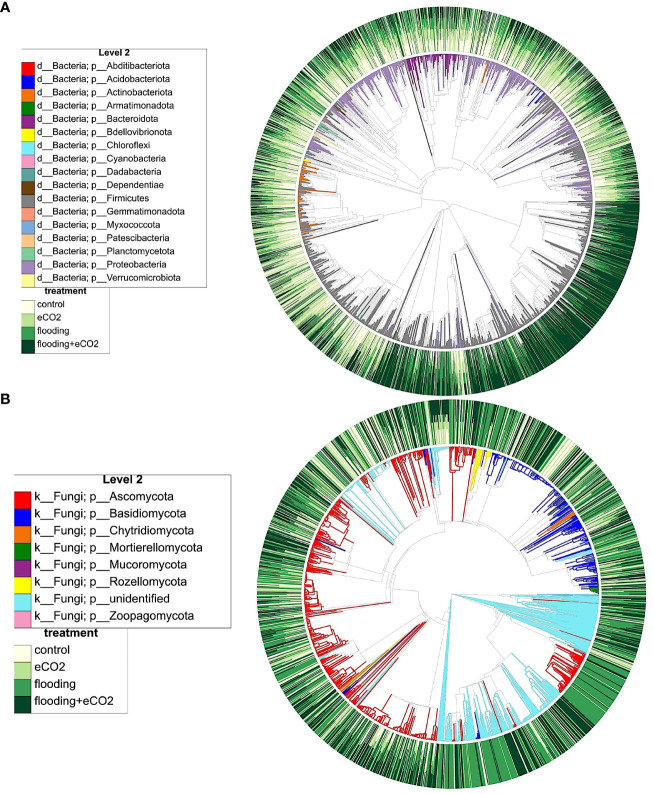
Phylogenetic clustering and interaction of different microbiome players from key phyla, their distribution during flooding, and eCO_2_ treatments. **(A)** shows the bacterial and **(B)** shows fungal phylogenetic clustering. The color distribution depicts the abundance pattern of OTUs across different treatments and their interactions. The outer circle shows the abundance levels (from light yellow to dark green), and the inner circle shows the dominance of specific microbiome players in different conditions.

The interactions of different microbiome species and clustering show that flooding and eCO_2_ strongly influence microbial species (**Figure 6**). For example, *Firmicutes* and *Proteobacteria* significantly cluster in response to both factors. A similar clustering was evident in the Ascomycota and Basidiomycota (**Figure 6**). To understand the taxa distribution and differential abundance in response to flooding and eCO_2_, we carried out ANCOM-BC2 (Lin and Peddada, 2020). The results showed that out of 288 taxa, 21 genera were differentially abundant in bacterial soil samples. Of the 21 genera, 20 taxa were differentially abundant in flooding + eCO_2_ stress (**Supplementary Table 11**; *p*< 0.05). While flooding stress only had one taxon of differential abundance, the family Lachnospiraceae. Increased eCO_2_ stress conditions had only two taxa of differential abundance: one from an uncultured genus and the other from Candidimonas.

Bacterial root samples had 15 taxa; only 7 were found differentially abundant. All seven differentially abundant taxa were present in flooding treatment, and all but two were in flooding + eCO_2_ treatment. The order Burkholderiales and the family Sphingomonadaceaea were differentially abundant in flooding but not in flooding + eCO_2_. The class Alphaproteobacteria was differentially abundant across all treatments except increased eCO_2_. Of the 13 taxa analyzed for differential abundance in bacterial shoot samples, only six were differentially abundant. eCO_2_ stress only had one taxon of differential abundance, the class Alphaproteobacteria, which is shared with the bacterial root samples. Two differentially abundant taxa were present in the flooding + eCO_2_ treatment: Pseudomonadaceae and Amphiplicatus. It is noted that Amphiplicatus was differentially abundant in both root and shoot bacterial ASVs for both flooding and flooding + eCO_2_ treatment (**Supplementary Table 11**).

In the case of fungal ASV, 16 of 95, 16 of 70, and 8 of 147 were found differentially abundant taxa in soil, root, and shoot samples, respectively. All but one taxon was differentially abundant in soil ITS samples for flooding treatment, except the genus *Epicoccum* which was differentially abundant in flooding + eCO_2_. Seven differentially abundant taxon for fungal shoot samples were from flooding + eCO_2_. Two genera were differentially abundant in flooding conditions, Plectosphaerella and Paecilomyces; the latter was also differentially abundant in flooding + eCO_2_. eCO_2_ treatment had one differentially abundant genus of *Fibulochlamys* (**Supplementary Table 12**).

### Influence of flooding and eCO_2_ on microbial enzymes in the rhizosphere

**Figure 7:**
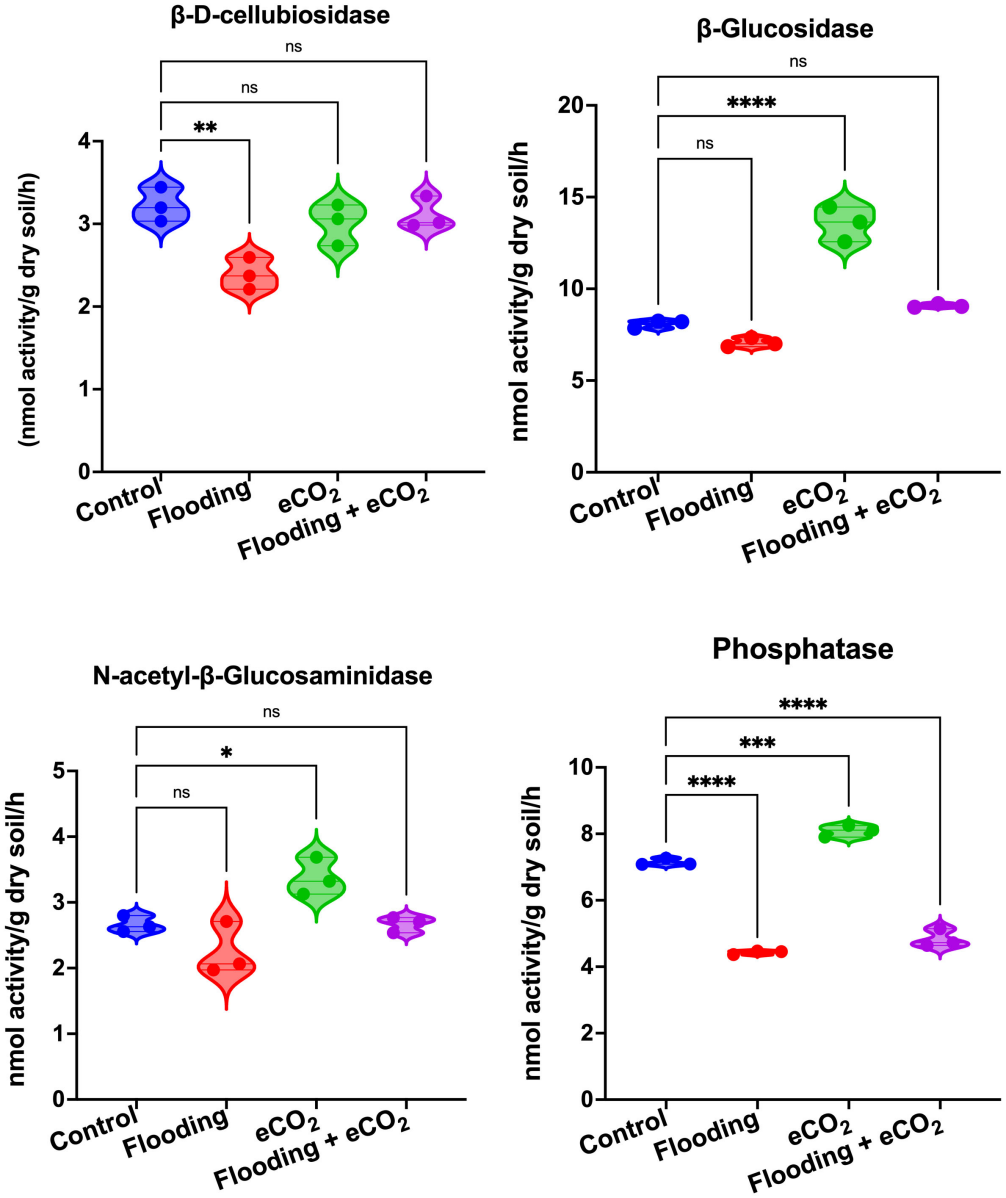
Extracellular enzymatic activities in rhizospheric soil of soybean plants treated with flooding and eCO_2_. The treatments were compared with the control (non-flooding). The values represent the mean values of three replicates and show standard deviation. The bars showing *, **, *** and **** are significantly different (p<0.05) in their content compared with the control as analyzed by two-way ANOVA analysis. “ns” shows that values are insignificant compared with control treatments.

We performed an analysis of the soil enzymes, viz., β-D-cellubiosidase (BDC), α-glucosidase (AG), β-glucosidase (BG), N-acetyl-β-glucosaminidase (NAG), and phosphatase (Phos), after eCO_2_, flooding, and eCO_2_ + flooding stress compared with non-flooded control. Our results showed that during flooding stress, the BDC activities were significantly (*p*< 0.05) reduced as compared with the control (**Figure 7**). In the case of eCO_2_ treatments with or without flooding, the BDC activities were non-significant compared with the control. The AG and BG enzymatic activities were non-significant during flooding stress (**Supplementary Figure 3**). However, among treatments, only the eCO_2_ application showed significantly (*p*< 0.001) higher activities of BG than AG compared with the control and other treatments (**Figure 7**). Overall, BG and AG showed lower enzyme activities during flooding stress. Phos enzyme activities were also significantly (*p*< 0.001) reduced during flooding stress compared with control soybean plants. Contrarily, the Phos activities were significantly (*p*< 0.001) increased by eCO_2_ compared with the control. Interestingly, these activities substantially reduced twofold in the combined treatment of flooding + eCO_2_ compared with the control (**Figure 7**).

**Figure 8:**
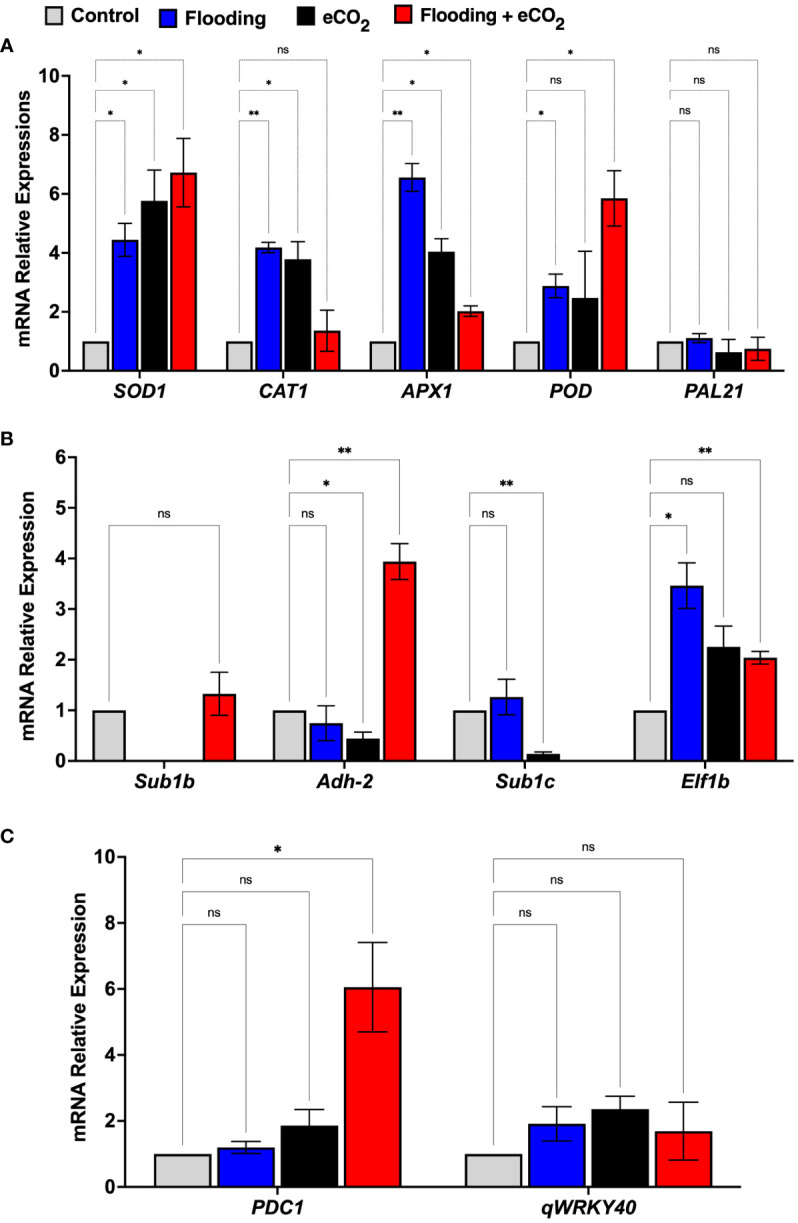
mRNA gene expression related to oxidative stress **(A)**, flooding **(B)**, and eCO_2_ **(C)** of soybean plants treated with flooding and eCO_2_. The treatments were compared with the control (non-flooding). The values represent the mean values of three replicates and show the standard deviation of relative expression to housekeeping genes and control. The bars showing * and ** are significantly different (p<0.05) in their content compared with the control as analyzed by two-way ANOVA analysis. “ns” shows that values are insignificant compared with control treatments.

To understand the molecular effect of CO_2_ and flooding stress, we investigated the relative expression of mRNA genes involved in CO_2_ and flooding stress and the oxidative defense system of soybean seedlings using qRT-PCR (**Figure 8**). The genes were chosen based on their relationship to oxidative defense, flooding, or elevated levels of CO_2_, with some being specific to *Glycine max*. Superoxide dismutase (*SOD1*), peroxidase (*POD*), catalase (*CAT1*), and ascorbate peroxidase (*APX*) are all oxidative defense genes that help reduce the damage of ROS during stress. Submergence-1b and -1c (*Sub1b* and *Sub1c*), alcohol dehydrogenase (*Adh-2*), and elongation factor 1 (*Elf-2b*) are genes related to flooding stress in plants. At the same time, pyruvate decarboxylase 1 (*PDC1*) catalyzes the first step in anaerobic fermentation.

The results showed that the relative expression of oxidative defense-related genes such as *SOD1* and *APX1* was significant (*p*< 0.001) in both the eCO_2_ and flooding stress alone and combined stress. The relative expression of the *SOD1* gene in flooding + eCO_2_ was the highest (6.72-fold) compared with flooding alone (4.4-fold), eCO_2_ alone (5.7-fold), and control. Similarly, the *POD* gene’s relative expression was higher in flooding + CO_2_ (5.8-fold) than in others (**Figure 8**). Interestingly, the *CAT1* gene expression was highly significant (6.5-fold) in flooding stress compared with eCO_2_ (4.03-fold), flooding + eCO_2_ (2.02-fold), and control. Furthermore, the flooding stress-associated genes were also investigated in which the *sub1b* gene was upregulated (1.3-fold) in combined flooding + eCO_2_ stress compared with control. Similarly, the *adh-2* gene showed the highest expression (3.9-fold) in combined flooding + eCO_2_ stress as compared with the control, whereas the *elf1b* gene showed the highest expression (3.4-fold) in flooding stress compared with eCO_2_ stress (2.2-fold), flooding + eCO_2_ (2.0-fold), and control. Interestingly, the *PDC1* gene, which is associated with eCO_2_ stress, showed the highest relative expression (6.0-fold) in combined flooding + eCO_2_ stress compared with the eCO_2_ (1.8-fold), flooding (1.19-fold), and control (**Figure 8**).

## Discussion

This study showed that flooding and eCO_2_ significantly impact the soybean plant growth attributes (shoot/root lengths and biomass) and photosynthetic pigments. In addition, these stress factors increase oxidative stress by regulating the antioxidant enzyme activities significantly compared with control or sole flooding and eCO_2_ treatments. Elevated CO_2_ alone did not show significant variation from the control except when considering the microbial activity. This study revealed that flooding in the presence of eCO_2_ influences the abundance of bacterial and fungal microbiome communities compared with control treatments and influences oxidative stress reactions. Changes in water level are a significant driver for plant growth and microbiome diversity. Previous studies showed that soybean is extremely sensitive to abiotic stress conditions (Trépanier, 2019; Longley et al., 2020). *Glycine max* is rich in oil and proteins (Hasanuzzaman et al., 2021). The flooding stress negatively affects its growth, development, and yield (Mustafa et al., 2015; Li et al., 2020). The resulting exudation of metabolites in the rhizosphere has also been argued for changes in symbiotic microbes (Sugiyama, 2019). The change in soil chemistry due to lack (drought) and abundance (high moisture) of water exacerbates the abundance of microbial communities (Jiao et al., 2023). Our results showed that flooding and eCO_2_ accelerated bacterial and fungal diversity.

Investigating further, we noticed a significant shift of microbial ASVs from the rhizosphere into the phyllosphere. Since both flooding and eCO_2_ created an abnormal growth condition, we propose a driving shift in the microbial community. Previous studies showed that plant cell division and gibberellic acid synthesis increase during flooding to escape hypoxia and expose the leaf to submergence (Kim et al., 2016). Microbial ASV abundances in the phyllosphere rather than in the rhizosphere suggest a similar phenomenon with microbial community structure.

We found significant variations across sole and combined treatments while looking at bacterial and fungal phylum distribution and its impact on their diversity due to flooding and eCO_2_. The family *Actinobacteria* was negatively impacted by soil moisture, while *Proteobacteria*, specifically *Betaproteobacteria* and *Gammaproteobacteria*, showed positive aggregation from soil moisture. In the case of eCO_2_, the microbial communities were not significantly affected compared with combined treatments. Microbiome richness across endophytic root bacterial and fungal communities appears resilient to the two factors. We showed that stress conditions increase bacterial richness in soil samples, but it caused a decrease in the endophytic root fungal community richness. The combined factors of flooding + eCO_2_ showed a significantly higher (*p*< 0.01) impact on microbial abundances. Some dominant bacteria phyla in flooding + eCO_2_ were *Bacteroidota*, *Firmicutes*, and *Proteobacteria.* Interestingly, we noticed a significant diversity of *Firmicutes* in the soil rhizosphere, but the same was significantly lower (*p*< 0.05) in the root endosphere. *Firmicutes* are known to be anaerobic species, which is likely why they play a large role during flooding stress (Martínez‐Arias et al., 2022). Contrarily, *Proteobacteria* were more abundant in flooding + eCO_2_, which are known to play a crucial role in abiotic stress environments (Vaishnav et al., 2018).

The phylum *Bacteroidota* remained stable in the soil rhizosphere during flooding + eCO_2_ stress but increased relative abundance due to flooding and eCO_2_ separately. Only two genera were found in the soil, root, and shoot samples: *Bacillus* and *Pseudomonas*. In addition, we found *Novosphingobium* sp., a rhizosphere-associated bacteria known to promote rice growth through N_2_ fixation and production of indole-3-acetic acid and siderophores in the rhizosphere (Krishnan et al, 2017; Vaishnav et al., 2018). Similarly, other dominant genera, such as *Sphingomonas* and *Bacillus*, have been previously shown to secrete gibberellins (Asaf et al., 2018). We hypothesize that a consortium of gibberellin-producing strains and their abundances in flooding + eCO_2_ could improve the plant cell division process in escaping the flooding condition. The plant growth-promoting characteristics, production of auxin and siderophores, and the solubilization of phosphate/silicate by bacteria can be key characteristics of plant stress tolerance in wheat (Moreira et al., 2016). These are also the key drivers of reshaping microbiome structure, as previously shown by Longley et al. (2020), where soil compositions lower microbial Shannon diversity.

Flooding and eCO_2_ also impacted fungal communities heavily, shifting the rhizosphere from *Basidiomycota*, *Ascomycota*, and unidentified to a loss of diversity, with *Ascomycota* almost completely dominating the biome. While the shift to the phylum *Ascomycota* occurred in flooding treatments irrelevant to eCO_2_ levels, the shift in the community during flooding with and without eCO_2_ levels varied significantly. We found three fungal genera upregulated in the rhizosphere during only flooding stress: *Didymella*, *Epicoccum*, and *Gibberella.* Of note, none of the previously mentioned fungal genera were significantly present with CO_2_ and flooding, with *Trichoderma* representing 90% of the fungal genera. The relative abundance of *Trichoderma* did not change when elevated CO_2_ levels were the only environmental factor compared with the control. *Trichoderma* is a well-known plant mutualist that offers a wide range of benefits to the host plant (Woo et al., 2023). The three genera discovered combined with flooding and eCO_2_ conditions are mostly known for their plant disease-causing species. For example, *Didymella* has been shown to cause leaf blight in maize and stem and leaf rot in legumes (Chen et al., 2015; Wille et al., 2019). *Gibberlla* and *Epicoccum* both have pathogenic species and others that can act as biological control agents. This information needs to be clarified as to whether these microbial shifts are solely caused by abiotic stress. It is more likely that an interplay of abiotic and biotic stressors will occur. A study has shown that when a plant experiences fungal infection, the plant can recruit beneficial genera (Gao et al., 2021). A network analysis would need to be performed to understand if what we see is pathogenic.

The phyllosphere sees the opposite shift where flooding induces microbial shifts, with the propagation of the *Basidiomycota* phylum. Combination stress of flooding and eCO_2_ increased genera. When eCO_2_ levels also occur, the unidentified fungal phylum is suppressed. A similar pattern to the rhizosphere in the phyllosphere can be seen where the fungal genera present have been studied and are seen to play both a pathogenic and beneficial role. *Didymella*, *Papilioterma*, and *Gibberella* are upregulated in the shoot of flooding and eCO_2_ stress. The line between pathogenic and beneficial is not easily elucidated due to the plant–microbe, plant–environment, and microbe–microbe feedback loops. The large presence of an *unidentified* fungal phylum represents a knowledge gap in our current fungal database. Research into the line between fungal pathogens and beneficiaries is emergent; as such, it is difficult to draw clear conclusions about the microbiome from this study alone. There was no evident sign of devastating infection upon plant harvest.

The shape of the microbiome correlates with the enzyme flux in the rhizospheric environment with soybean plants. The soil enzymes BDC, AG, BG, NAG, and Phos showed significant reduction after flooding and eCO_2_ + flooding stresses compared with control. Studies have shown that high microbial activities in the rhizosphere often correspond to increased activities of enzymes. This also correlates to the lower microbiome diversity in the soil part during stress conditions. However, BG, NAG, and Phos were significantly higher in eCO_2_ than in other treatments. These have been recently correlated with high β-diversity in the rhizosphere of wheat plants (Jin et al., 2022). A recent study showed that Phos directly correlates to the relative abundances of Bacteroidetes, Gemmatimonadetes, and *Funneliformis* in bacterial and fungal communities, respectively (Jin et al., 2022). We show that elevated CO_2_ does not mitigate the negative impacts that flooding has on rhizospheric microbial activity. The mechanisms of enzymatic activities and the influence of eCO_2_ have not been fully explored. Due to rising CO_2_ levels, investigating the role of carbon dioxide in these mechanisms is imperative.

At the same time, the soybean root-secreted metabolites play a pivotal role in shaping the microbial community structure in the rhizosphere (Sugiyama, 2019). Isoflavonoids are prominent rhizodeposits in soybean that help defend and enable symbiotic associations with rhizobia (White et al., 2017). Daidzein and genistein are isoflavonoids produced by soybean into the rhizosphere to communicate with rhizobia, establish nodulation, and play a role in defense against pathogens (Ng et al., 2011). In soybean, *Bradyrhizodium* and *Gammaproteobacteria* (*Proteobacteria* phylum) were dominant and associated with crop productivity during abiotic stresses (Chang et al., 2017). Similarly, *Actinobacteria*, *Chloroflexi*, *Proteobacteria*, *Ascomycota*, *Basidiomycota*, and *Mortierellomycota* phyla were significantly dominant in the soybean that was grown in different soil textures (Trépanier, 2019).

Little is known about the potential function of the single microbial family playing a dominant role during flooding stress. However, *Klebsiella variicola* and *Azospirillum* sp. were isolated and improved plant growth during flooding by forming adventitious roots in soybean plants (Kim et al., 2017; Tiwari et al., 2020). Due to flooding, there are more chances that the soil O_2_ levels are quickly depleted by aerobic microbes, reaching anoxia even in the uppermost bulk soil layers within hours of a flooding event. This change in O_2_ availability can then result in a progressive shift in the microbial community from aerobic organisms to facultative anaerobes and finally to strict anaerobes (Shabala et al., 2014). This shift toward anaerobic bacteria was hypothesized to be one possible explanation behind the increase in the relative abundance of *Aquaspirillum* in flooded poplar rhizosphere and root samples, as the genus contains a few known anaerobic species (Graff and Conrad, 2005). They hypothesized that shifts in the denitrifying bacterial community resulted from the combined effects of O_2_ and N stress on the plant, which can reduce root C exudation. Although some evidence supports altered exudation of total organic carbon in plants exposed to flooding, changes in root exudates from flooded non-wetland species and consequent effects on root microbial communities remain relatively unexplored (Tiwari et al., 2020).

Under flooding and elevated levels of CO_2_ stress, a higher amount of ROS is produced in various components of plant cells, disrupting normal plant metabolism (Jabeen et al., 2020; Lubna et al., 2022). Typically, ROS are formed when the electrons (one, two, or three) are transferred to molecular oxygen (O_2_^−^), which results in hydroxyl (OH), hydrogen peroxide (H_2_O_2_), or superoxide (O_2_^−^) radicals (Bhattacharjee, 2010). To survive, plants activate the antioxidant defense system to mitigate oxidative damage (Hasanuzzaman et al., 2021). To alleviate flooding and eCO_2_ stress-generated ROS, plants accelerate the production of antioxidant defense systems like SOD, POD, and other non-enzymatic antioxidants (Hasanuzzaman et al., 2021). SOD mediates the detoxification of superoxide radicals and prevents stress-induced cellular damage (Hasanuzzaman et al., 2021). Flooding stress induces interesting changes in gene expression, which coordinate morphological and metabolic adaptations to stress. The *SOD*, *APX*, and *POD* genes showed elevated expression in flooding and eCO_2_ stresses alone and combined, as reported previously in *Luffa aegyptiaca* under flooding stress (Chiang et al., 2014) and durum wheat under eCO_2_ stress (Medina et al., 2016). This indicates that elevated CO_2_ in the presence of flooding might change the biochemical pathways soybeans use to cope with stress. This is also supported by the fact that catalase antioxidant was significantly downregulated in the stem compared with only flooding treatment. Although the change was not statistically significant, there was a slight decrease in *PPO* antioxidants, mostly in both the root and leaf, when elevated CO_2_ was in conjunction with flooding treatments. Furthermore, the *Sub1* gene family regulates submergence tolerance in flooding stress. We presume that eCO_2_ can be consumed by several classes of microbes and plant roots as a carbon source. This may lead to a reduction in oxidative stress in the root region. Alternatively, the weak carbonic acid and related radicals can react with flooding-induced radicals to develop a cascade of reactants and products to reduce oxidative stress in the rhizosphere. These genes enhance tolerance by minimizing the ethylene-promoted GA responsiveness by enhancing the accumulation of the GA signaling. In the current study, the *Sub1* gene was upregulated compared with control plants, and the expression patterns increased due to flooding stress (Fukao et al., 2019).

## Conclusion

It has been noted that increasing our mechanistic understanding and real-world understanding of microbiome–plant interactions under flooding stress offers enormous potential for increasing the resilience of plants in such conditions (Van Der Heijden and Hartmann, 2016; De Vries et al., 2020). This topic is becoming essential as climatic risk events such as flooding and drought influence agricultural productivity. While we have studied flooding and its effect on plants, minimal studies consider the rising levels of CO_2_ in our atmosphere. This is relevant because flooding creates hypoxic conditions that elevated CO_2_ has the potential to worsen. Hence, we show that flooding and eCO_2_ drastically impact plant growth physiology, gene expression profiling, and phenotype. Our findings show that these biochemical changes must be investigated further to understand the effect of flooding and eCO_2_ on the holobiont. Elevated CO_2_ levels reduce microbial activity in the soil, and the role it plays in the plant’s biosynthetic pathways is not clear, opening up opportunities for future research investigating this. These environmental stressors, either alone or in combination, significantly impact the diversity and abundance of bacterial and fungal communities. Current fungal databases are missing an important fungal phylum identification that has the potential to play a critical role in soybean health and disease. Our findings highlight potential knowledge gaps in microbiome–plant relationships.

## Materials and methods

### Plant material, growth conditions, and treatment

*Glycine max* L. (Fiskeby III soybean) obtained from the US Department of Agriculture was selected due to its ability to show resistance against abiotic stresses. The soybean seeds were germinated in a soil mixture of peat moss (Miracle-Grow, USA), organic topsoil, and Ferti-lome perlite in 40:30:10 ratios, respectively. The soil mixture was thoroughly mixed and autoclaved to induce the development of the native microbiome in sterile conditions. A recent study has shown that soil disruption via autoclaving increased the colonization on the rhizosphere of potentially beneficial bacterial genera by reducing the number of microbial pathogens present and that these bacteria are shown to be crop-specific potentially (Dilegge et al., 2022). While this method may not present accurate agricultural settings, it will allow us to screen for genera specific to our plant of interest. This will allow us to find soybean-specific potential growth-promoting bacteria or fungi. It is customary to include bulk soil analysis; unfortunately, we could not collect samples and use the control as a reference for changes in this study. The plants were grown till the V3 stage in a growth chamber (Biora, MineARC Sys Inc., USA; relative humidity 60%–70% and light intensity of 800 μE m^−2^ s^−1^ from sunlight Z4NW; day/night cycle of 14 h at 28°C and 10 h at 25°C). The pots were watered with autoclaved DI water (ADW). After stage V3, the plants were arranged in a fully factorial experimental design with two factors: i) flooding and ii) eCO_2_ treatments. Thus, it was comprised of i) control, ii) flooding, iii) eCO_2_, and iv) flooding + CO_2_. The control plants received only DW to maintain a natural soil moisture level of 50%. The flooding stress was induced by exposing plants to submergence for 7 days at 7 inches above the soil surface (partial submergence). An eCO_2_ stream was applied every 12 h to maintain an eCO_2_ level of 680 ± 80 ppm for 7 days with or without flooding stress. The eCO_2_ levels were monitored using a sensor (Vaisala, Helsinki, Finland). Each treatment comprised 21 plants which were all harvested 7 days after the beginning of treatments. After 7 days of treatments, the different plant growth parameters (plant length, biomass, and chlorophyll contents) were taken. The plant and soil samples were harvested with gloves being used, and the plants were removed from pots. The soil surrounding the roots was shaken into an ethanol-cleaned bin and collected for soil samples. The roots were then rinsed in water to remove the remaining soil particles. The plant biomass was weighted and the roots and stems were cut to be ground in liquid nitrogen separately. After grinding the samples with liquid nitrogen, they were kept at −80°C until further analysis.

### Plant growth and oxidative stress analysis

Plant growth attributes, including shoot and root length and biomass, were recorded. Chlorophyll content, total nitrogen, and leaf surface humidity were measured using a chlorophyll meter (Minolta, Japan). For a detailed analysis, chlorophyll (*a*, *b*, and total), carotenoids, and flavonoids were analyzed via spectrophotometry (Imran et al., 2021). Oxidative stress enzymes (superoxide anions and H_2_O_2_) were also analyzed for all the treatments. Leaf and root samples were ground to a fine powder, and a 0.2-g subsample was used for each extraction. Superoxide anions were extracted with 5 ml of buffer [25 ml of 10 mM phosphate buffer pH 7.8 + 15 ml of 0.05% nitroblue tetrazolium chloride (NBT) + 10 ml of 10 mM NaN_3_]. The samples were incubated for 30 min at room temperature with shaking and were then incubated in a water bath at 70°C for 15 min. After cooling to room temperature, the samples were centrifuged at 10,000 rpm for 15 min. The supernatant was taken at a volume of 250 μl and added to 96-well plates to be read at 580 nm absorbance (Khan et al., 2020a). To determine the hydrogen peroxide (H_2_O_2_) level, 10% trichloroacetic acid (TCA) was added to the samples. The samples were vortexed and then centrifuged at 4,000 rpm for 10 min. The supernatant was collected, and 50 μl was added to a 96-well plate. Then, 100 μl of 1 M potassium iodide and 50 μl of 10 mM phosphate buffer were added to all the wells. The absorbance was read at 390 nm. TCA was added to the samples for reduced glutathione 5 ml of 10%. The samples were vortexed and then centrifuged at 4,000 rpm for 10 min. The collected supernatant reacted with Ellman’s reagent in the presence of a phosphate buffer (pH 6.8; 100 mM). The plate was read at 420 nm absorbance. Similarly, catalase, polyphenol oxidase, peroxidase, and superoxide dismutase were analyzed using an extraction buffer (30 mM Tris–HCl + 6 mM MgCl_2 +_ 1 mM EDTA + 3.5 PVP). Samples were vortexed and centrifuged (4,000 rpm for 10 min at 4°C). The supernatant was used for catalase reaction (50 μl of supernatant, 150 μl of 10 mM phosphate buffer (pH 6.8) + 50 μl of 0.2 M H_2_O_2_) that was read on a spectrophotometer (Tecan 10M; at 240 nm, 255 nm, and 280 nm; Aebi, 1984). For polyphenol oxidase, the supernatant (50 μl) was mixed with 50 μl of pyrogallol (50 μM) and 100 μl of phosphate (pH 6.8; 100 mM). The plate was read at 420 nm absorbance. For peroxidase, the 50-μl supernatant was mixed with 50 μl of pyrogallol (50 μM), 25 μl of H_2_O_2_ (50 μM), and 100 μl of phosphate buffer (100 mM, pH 6.8) and read using a spectrophotometer (Tecan 10M; at 420 nm). All the experiments were performed in triplicates (Khan et al., 2020a).

### Extracellular enzyme analysis

A solution of MUB (4-methylumbelliferone, 1 μM) in sodium acetate (pH 5.2) buffer was used as the fluorescent substrate. Testing was performed according to previous protocols from both Marx et al and Jian et al (Marx et al., 2005; Jian et al., 2016). Exozymes used in the study were β-D-cellubiosidase (BDC), α-glucosidase (AG), β-glucosidase (BG), N-acetyl-β-glucosaminidase (NAG), and phosphatase (Phos). Each exozyme used was quantified on the fluorescence spectrophotometer (Shimadzu, Tokyo, Japan). The rhizospheric soil samples from all treatments were incubated in sodium acetate buffer (pH 5.2) for 24 h on shaking (150 rpm). The samples were centrifuged (4°C, 12,000 rpm for 20 min), and resulting supernatants were collected. If turbidity was present a 0.22 mm filtered syringe was used. Five replicates for each substrate were taken per enzyme analysis. The samples were run on the same machine following exozyme quantification. Readings were taken at absorbance 360 nm and 460 nm for excitation and emission respectively at times 0 and 30 minutes. The concentrations were calculated in μmol h^−1^ L^−1^ (Stroud et al., 2022).

### Microbiome DNA extraction and analysis

The samples (rhizospheric soil, root, and shoot) were harvested from four treatments—i) control, ii) flooding, iii) eCO_2_, and iv) flooding + eCO_2_—after the stress conditions. The plant tissues were processed according to Mcpherson et al. (2018). The leaves and roots were ground into a fine powder using a mortar and pestle using liquid nitrogen. The MagMAX™ Plant DNA Kit (Thermo Scientific, Massachusetts, USA) was used to extract DNA from plant leaves and roots. The manufacturer’s instructions were used with a few modifications to extract high molecular weight DNA. A modified method (Verma et al., 2017) was used to extract soil DNA. Briefly, soil (0.2 g) samples were suspended in 1.4 ml of extraction buffer [100 mM of Tris/HCl (pH 8.0), 100 mM of EDTA (pH 8.0), 100 mM of sodium phosphate buffer (pH 8.0), 1.5 M of sodium chloride, 1% (w/v) CTAB, 100 mM of calcium chloride, 100 mg of lysozyme/ml]. The soil slurry was incubated at 37°C for 1 h with shaking at 200 rpm. Following incubation, 0.3 ml of SDS (20%) was added and incubated at 65°C for 1 h in a water bath with shaking every 10 min. The samples were centrifuged at 7,000*g* for 20 min at 4°C, and the supernatant was collected. Equal volumes of chloroform:isoamyl alcohol (24:1) were added to the supernatant and then centrifuged at 14,000*g* for 20 min at 4°C. The samples were kept on ice after the addition of chloroform:isoamyl alcohol. The top aqueous phase was collected, and 0.1 volume of 3 M sodium acetate and 0.4 volume of 30% PEG-8000, w/v, were added and then incubated at −20°C for 45 min. The samples were again centrifuged at 14,000*g* for 15 min at 4°C. The supernatant was discarded, and the pellet was dissolved in 70% ethanol. After dissolving in ethanol, the samples were centrifuged at 14,000*g* for 15 min at 4°C with the supernatant discarded. After drying, the pellets were resuspended in 60 μl of nuclease-free water. The quality and purity of all DNA samples were checked using a Thermo Scientific NanoDrop Lite Spectrophotometer (Massachusetts, USA) and an Invitrogen™ Qubit™ 4.0 Fluorometer (California, USA).

### Microbiome sequencing

The DNA was processed for amplicon sequencing. PCR-free libraries of each DNA sample were generated by amplifying the internal transcribed spacers (ITS2 and ITS4) and 16S rRNA (V3–V4) for fungal and bacterial communities, respectively. For 16S rRNA, peptide nucleic acid (PNA) clamps were used to reduce mitochondrial and chloroplast contamination. A paired-end sequencing approach of 300 bp was conducted on an Illumina MiSeq instrument (Illumina Inc., San Diego, CA, USA) operating with v2 chemistry (User Guide Part # 15,027,617 Rev. L). All quality reads related to the study are available at NCBI under BioProject (PRJNA875044), BioSample (SAMN30594393), and accession number (SRR23345057–SRR23345080).

### Bioinformatics analysis

The sequencing reads were analyzed with QIIME2.0 (Bolyen et al., 2019). The read quality was assessed with fast QC. We used the Mothur and DADA2 algorithms for denoising and generating the amplicon sequence variants (ASVs) (Callahan et al., 2016). In the denoising, sequences were filtered by overall quality and trimmed in low-quality regions, and chimeric sequences were removed (Callahan et al., 2017a). The 16S rRNA gene reads were trained on the SILVA database for the taxonomic classification (Quast et al., 2012), while the UNITE database was used to classify the ITS sequences (Nilsson et al., 2019). Sequences classified as mitochondria and chloroplast were removed from the 16S rRNA gene ASV table. For beta-diversity analyses, the Bray–Curtis distance and unweighted UniFrac PCoA matrix were generated for the sequence dataset and exported to RStudio software for statistical analysis. The Shannon diversity index and the observed ASV richness were calculated for alpha-diversity analyses. Permutative multivariate analysis of variance (PERMANOVA, 999 permutations) was used to test for significant effects of the factors (plant compartment, flooding, and eCO_2_) and their interaction on bacterial and fungal community composition using the “adonis function.” ANCOM-BC2 (Lin and Peddada, 2020) and analysis of similarity (ANOSIM) were also used to test the effects of the factors on the fungal and bacterial communities using RStudio. Differences in species diversity (Shannon index) and richness (observed ASVs) for the same factors were assessed using the Kruskal–Wallis test in QIIME 2.0 (Bolyen et al., 2019). The DESeq2 package was used to implement a negative binomial generalized model to test the effect of eCO_2_ and flooding on the ASV abundances.

### Molecular gene expression analysis

High molecular weight RNA was extracted from the aerial (shoot/leaf) samples using the MagMAX™ Plant RNA Isolation Kit (Thermo Fisher Scientific, Massachusetts, USA). The extracted RNA was analyzed for quantity and integrity through Qubit 4.0 (Qubit RNA IQ Assay and RNA HS Assay kits; Thermo Fisher Scientific, Massachusetts, USA). The cDNA synthesis was performed using the standard protocol of the kit (High Capacity cDNA Reverse Transcription; Applied Biosystems, California, USA). RNA (10 µl and 100 ng/µl) was added to the master mix, and cDNA was synthesized through polymerase chain reaction (PCR) in a thermocycler under specific conditions (25°C for 10 min, 37°C for 2 h, and 85°C for 5 min). The synthesized cDNA was stored at −80°C until further use. The synthesized cDNA was normalized and used for gene amplification. Power up “SYBR” green Master Mix has been used for the thermocycler (QuantStudio 7 Pro Flex, Applied Biosystems, California, USA) PCR reaction. Primers ordered from Azenta (forward and reverse) were used at 10 pM for all reactions (**Supplementary Table 12**). The qPCR reaction conditions were 94°C for 10 min, followed by 35 cycles of 94°C for 45 s, 65°C for 45 s, and 72°C for 1 min, and a final extension at 72°C for min. Gene expression results were analyzed using delta CT calculation methods, and the experiment was repeated three times. Fold changes in gene expression were calculated using the formula described previously (Khan et al., 2021).

### Statistical analysis

At least three replicates per treatment were analyzed during this study. The data for the enzyme study are presented as the mean ± standard error (SEM). The significant differences were determined using a two-way analysis of variance (ANOVA). The two factors, flooding and eCO_2_, were considered and computed across treatments to know the significance level. The mean values were considered significant at *p<* 0.05 and were calculated by GraphPad Prism Version 9.01 (GraphPad Software, San Diego, CA, USA).

## Data availability statement

The original contributions presented in the study are included in BioProject (PRJNA875044), BioSample (SAMN30594393), accession number (SRR23345057–SRR23345080) and in the article/**Supplementary Material**, further inquiries can be directed to the corresponding author/s.

## Author contributions

LC: Formal analysis, Methodology, Software, Visualization, Writing – original draft. HM: Methodology, Writing – review & editing. YA: Investigation, Methodology, Writing – review & editing. RM: Investigation, Methodology, Writing – review & editing. WA: Software, Validation, Visualization, Writing – review & editing. KC: Resources, Writing – review & editing. AK: Conceptualization, Funding acquisition, Methodology, Project administration, Supervision, Writing – review & editing, Investigation.
