# Supplementary material for: Microbiome structure variation and soybean’s defense responses during flooding stress and elevated CO2
Source: Front Plant Sci. 2024 Feb 8;14:1295674. doi: 10.3389/fpls.2023.1295674 (PMC10882081; doi:10.3389/fpls.2023.1295674)
Supplement: Supplementary file 1 [file DataSheet_1.docx]

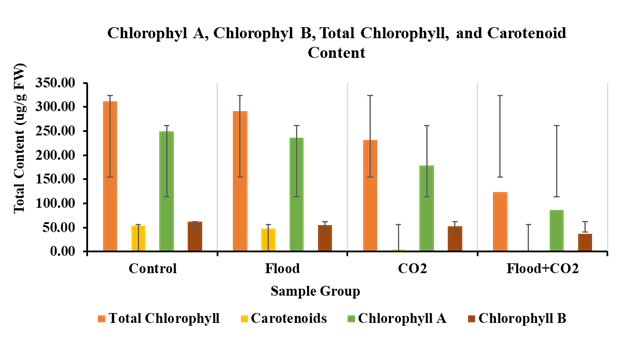


**Fig S1:** Effect of flooding and CO2 treatment on the photosynthetic pigmentation of plants.


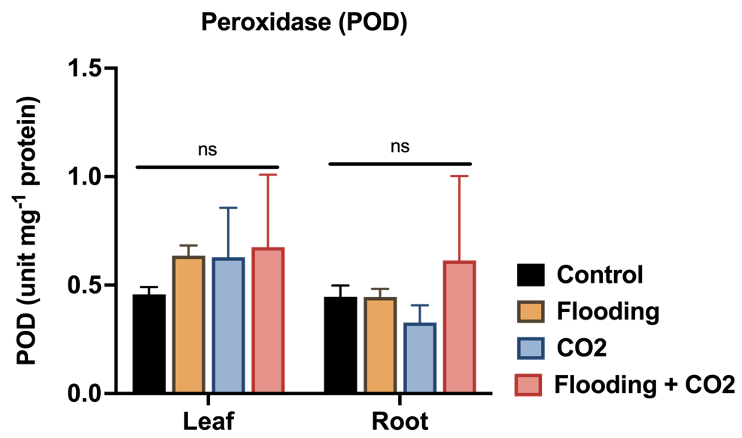


**Fig S2**: Peroxidase enzyme activities in soybean plants after Flooding and CO2 application.
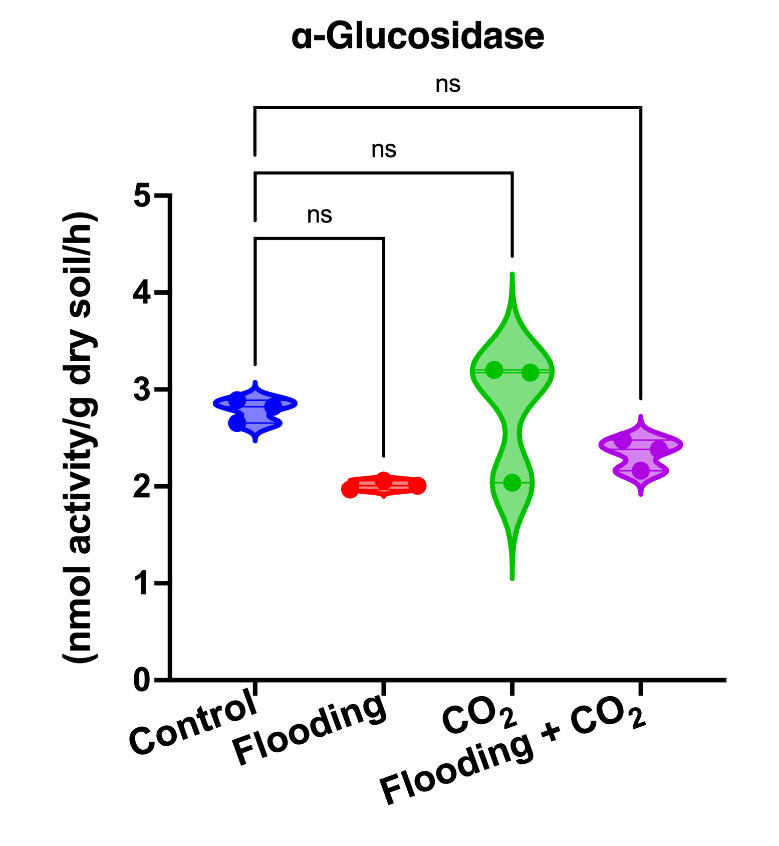


**Fig S3:** α-Glucosidase extracellular enzyme activities in soybean plants after Flooding and CO2 application.


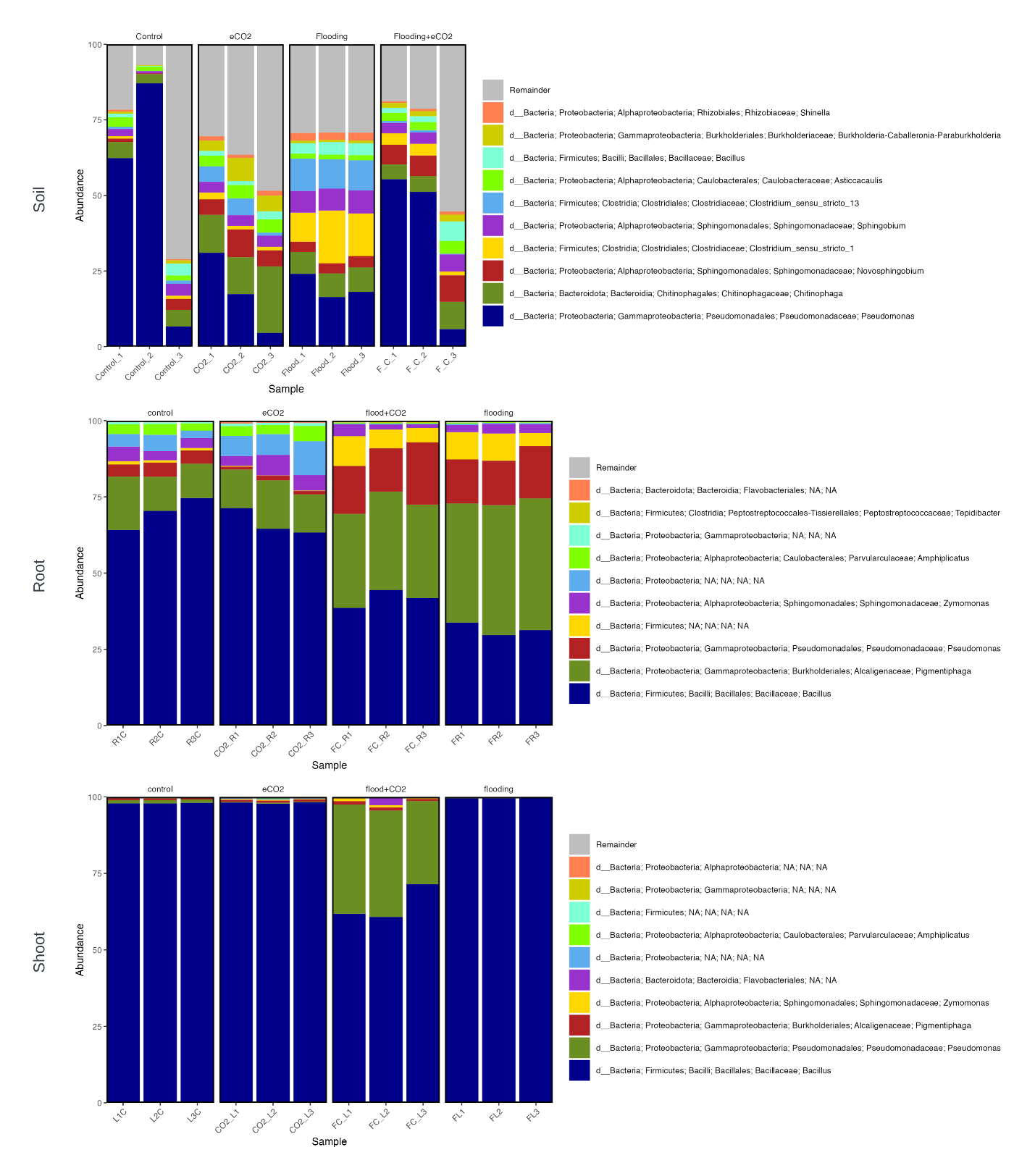


**Fig S4:** Taxonomical bar plots of genus level bacterial relative abundance for 16S samples


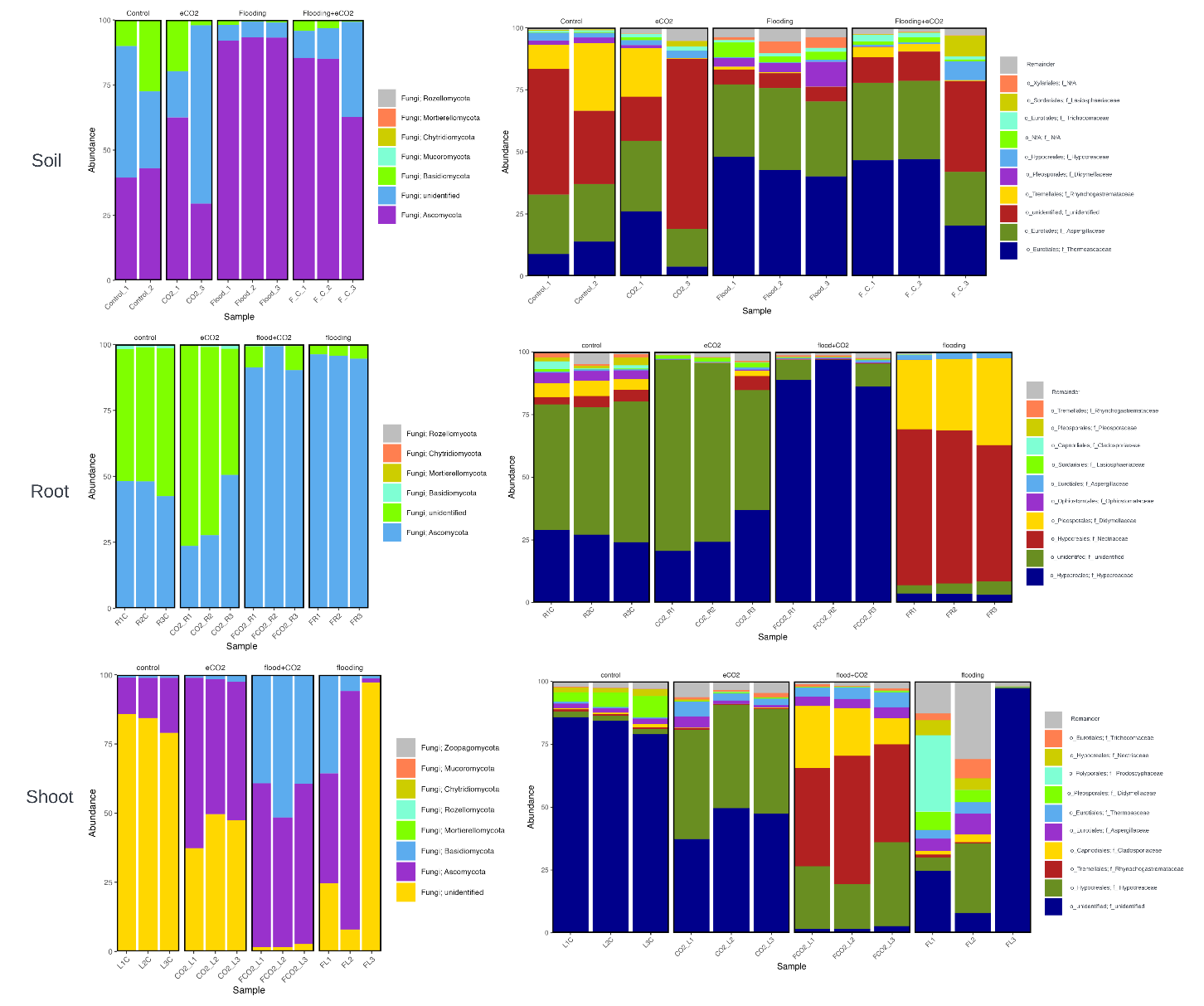


**Fig S5:** Taxonomical bar plots showing Phylum level represented on the left and family level represented on the right. The top shows Soil, middle Root, and bottom Shoot of fungal samples


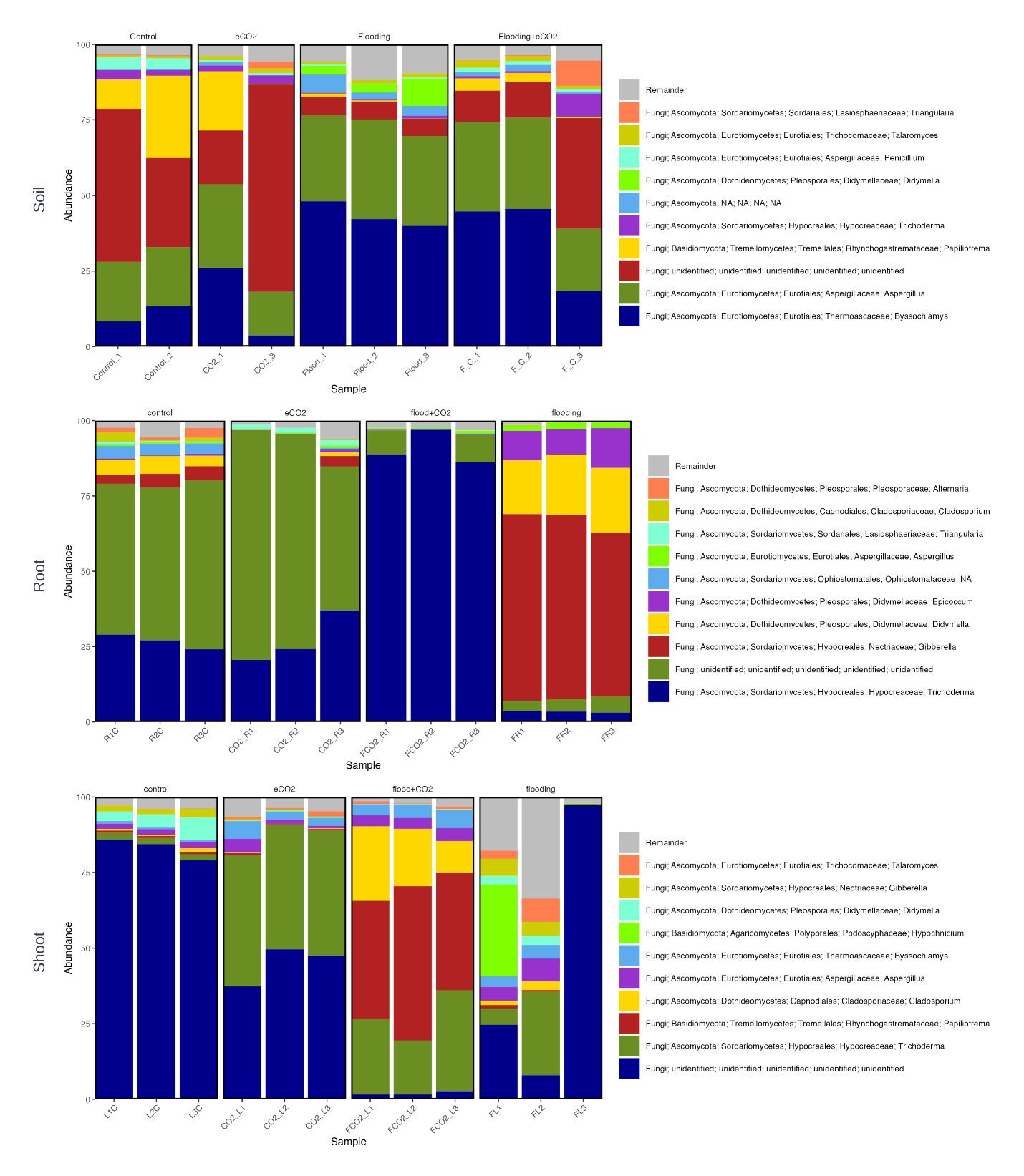


**Fig S6:** Taxonomical bar plots of genus level fungal relative abundance for ITS samples

**Table S1:** Plant growth attributes of different treatments compared to control soybean plants.

|  | **Control (1)** | **Flood and CO2** | **CO2** | **Flood** |
| --- | --- | --- | --- | --- |
| **Leaves (#)** | 10 | 4 | 4 | 13 |
| **Shoot (in.)** | 6.25 | 7 | 6.75 | 17.5 |
| **Root (in.)** | 9.5 | 6 | 9 | 8 |
| **Internode (in.)** | 2.25 | 2.25 | 2.75 | 2.4 |
| **Biomass (g)** | 4.2 | 1.8 | 2.0 | 2.6 |
| **Chlorophyll (SPAD)** | 45.7 | 47.75 | 38.9 | 22.9 |
| **pH** | 5.54 | 5.84 | 5.62 | 5.74 |

**Table S2:** Novogene filtering and quality control F001, 16S soil samples.

| **Sample** | **NovoID** | **Raw Reads** | **Clean Reads** | **Raw Base (G)** | **Clean Base (G)** | **Error Rate (%)** | **Q20 (%)** | **Q30 (%)** | **GC Content (%)** |
| --- | --- | --- | --- | --- | --- | --- | --- | --- | --- |
| Control_3 | FKDN220186460-1A | 163795 | 163739 | 0.08 | 0.08 | 0.03 | 97.07 | 91.73 | 52.6 |
| Control_2 | FKDN220186459-1A | 183047 | 183047 | 0.09 | 0.09 | 0.03 | 97.04 | 91.57 | 52.79 |
| CO2_1 | FKDN220186440-1A | 177887 | 177872 | 0.09 | 0.09 | 0.03 | 97.15 | 91.87 | 51.7 |
| Control_1 | FKDN220186458-1A | 161226 | 161218 | 0.08 | 0.08 | 0.03 | 96.94 | 91.53 | 52.84 |
| Flood_3 | FKDN220186451-1A | 176631 | 176628 | 0.09 | 0.09 | 0.03 | 97.19 | 92.01 | 51.98 |
| Flood_2 | FKDN220186450-1A | 174015 | 174011 | 0.09 | 0.09 | 0.03 | 97.11 | 91.85 | 51.88 |
| Flood_1 | FKDN220186449-1A | 174799 | 174792 | 0.09 | 0.09 | 0.03 | 97.16 | 91.95 | 52.15 |
| CO2_3 | FKDN220186442-1A | 180298 | 180283 | 0.09 | 0.09 | 0.03 | 97.21 | 91.94 | 52.26 |
| CO2_2 | FKDN220186441-1A | 178807 | 178807 | 0.09 | 0.09 | 0.03 | 97.39 | 92.19 | 52.71 |
| F_C_2 | FKDN220186432-1A | 186485 | 186485 | 0.09 | 0.09 | 0.03 | 97.22 | 91.96 | 52.51 |
| F_C_1 | FKDN220186431-1A | 169697 | 169697 | 0.08 | 0.08 | 0.03 | 97.04 | 91.56 | 52.61 |
| F_C_3 | FKDN220186433-1A | 169962 | 169958 | 0.08 | 0.08 | 0.03 | 97.22 | 91.89 | 52.72 |

**Table S3**: Novogene filtering and quality control F002, 16S shoot and root samples.

| **Sample** | | **NovoID** | | **Raw Reads** | **Clean Reads** | **Raw Base (G)** | **Clean Base (G)** | **Error Rate (%)** | **Q20 (%)** | **Q30 (%)** | **GC Content (%)** |
| --- | --- | --- | --- | --- | --- | --- | --- | --- | --- | --- | --- |
| FCO2_R1 | | FKDN220186425-1A | 170609 | 170606 | 0.09 | 0.09 | 0.03 | 96.77 | 91.22 | 52.83 |  |
| FCO2_R2 | | FKDN220186426-1A | 171035 | 171031 | 0.09 | 0.09 | 0.03 | 96.69 | 90.93 | 52.85 |  |
| FCO2_R3 | | FKDN220186427-1A | 179599 | 179595 | 0.09 | 0.09 | 0.03 | 96.91 | 91.55 | 52.89 |  |
| FCO2_L1 | | FKDN220186428-1A | 161978 | 161977 | 0.08 | 0.08 | 0.03 | 97.2 | 92.18 | 52.8 |  |
| FCO2_L2 | | FKDN220186429-1A | 183482 | 183481 | 0.09 | 0.09 | 0.03 | 97.18 | 92.13 | 52.68 |  |
| FCO2_L3 | | FKDN220186430-1A | 171701 | 171700 | 0.09 | 0.09 | 0.03 | 97.15 | 92.07 | 52.77 |  |
| CO2_R1 | | FKDN220186434-1A | 182119 | 182117 | 0.09 | 0.09 | 0.03 | 97.1 | 91.88 | 53.52 |  |
| CO2_R2 | | FKDN220186435-1A | 181668 | 181666 | 0.09 | 0.09 | 0.03 | 97.13 | 92 | 53.83 |  |
| CO2_R3 | | FKDN220186436-1A | 183575 | 183575 | 0.09 | 0.09 | 0.03 | 96.97 | 91.55 | 53.42 |  |
| CO2_L1 | | FKDN220186437-1A | 167928 | 167928 | 0.08 | 0.08 | 0.03 | 97.46 | 92.74 | 53.58 |  |
| CO2_L2 | | FKDN220186438-1A | 151021 | 151020 | 0.08 | 0.08 | 0.03 | 97.52 | 92.82 | 53.56 |  |
| CO2_L3 | | FKDN220186439-1A | 166459 | 166458 | 0.08 | 0.08 | 0.03 | 97.48 | 92.76 | 53.59 |  |
| FR1 | | FKDN220186443-1A | 169295 | 169294 | 0.08 | 0.08 | 0.03 | 97.02 | 91.85 | 52.38 |  |
| FR2 | | FKDN220186444-1A | 187806 | 187803 | 0.09 | 0.09 | 0.03 | 97.05 | 91.86 | 52.23 |  |
| FR3 | | FKDN220186445-1A | 186798 | 186797 | 0.09 | 0.09 | 0.03 | 96.8 | 91.4 | 52.55 |  |
| FL1 | | FKDN220186446-1A | 158426 | 158426 | 0.08 | 0.08 | 0.03 | 97.54 | 92.84 | 53.63 |  |
| FL2 | | FKDN220186447-1A | 189531 | 189530 | 0.09 | 0.09 | 0.03 | 97.52 | 92.83 | 53.59 |  |
| FL3 | | FKDN220186448-1A | 162967 | 162966 | 0.08 | 0.08 | 0.03 | 97.43 | 92.72 | 53.6 |  |
| R1C | | FKDN220186452-1A | 170404 | 170393 | 0.09 | 0.09 | 0.03 | 96.85 | 91.38 | 53.64 |  |
| R2C | | FKDN220186453-1A | 165551 | 165539 | 0.08 | 0.08 | 0.03 | 96.99 | 91.63 | 53.3 |  |
| R3C | | FKDN220186454-1A | 171072 | 171059 | 0.09 | 0.09 | 0.03 | 97.09 | 91.91 | 53.42 |  |
| L1C | | FKDN220186455-1A | 152392 | 152392 | 0.08 | 0.08 | 0.03 | 97.48 | 92.75 | 53.57 |  |
| L2C | | FKDN220186456-1A | 182280 | 182279 | 0.09 | 0.09 | 0.03 | 97.5 | 92.78 | 53.54 |  |
| L3C | | FKDN220186457-1A | 160649 | 160649 | 0.08 | 0.08 | 0.03 | 97.44 | 92.67 | 53.55 |  |

**Table S4**: Novogene filtering and quality control F005, ITS soil samples.

| **Sample** | **NovoID** | **Raw Reads** | **Clean Reads** | **Raw Base (G)** | **Clean Base (G)** | **Error Rate (%)** | **Q20 (%)** | **Q30 (%)** | **GC Content (%)** |
| --- | --- | --- | --- | --- | --- | --- | --- | --- | --- |
| Flood_3 | FKDN220186451-1A | 188670 | 176116 | 0.09 | 0.09 | 0.04 | 95.85 | 89.83 | 57.08 |
| F_C_1 | FKDN220186431-1A | 175692 | 173707 | 0.09 | 0.09 | 0.03 | 95.17 | 88.65 | 59.38 |
| F_C_3 | FKDN220186433-1A | 169656 | 168298 | 0.08 | 0.08 | 0.03 | 95.55 | 89.6 | 53.79 |
| Flood_2 | FKDN220186450-1A | 161623 | 151315 | 0.08 | 0.08 | 0.03 | 96.81 | 92.15 | 57.45 |
| Control_1 | FKDN220186458-1A | 188754 | 188217 | 0.09 | 0.09 | 0.03 | 96.56 | 91.46 | 51.19 |
| F_C_2 | FKDN220186432-1A | 186689 | 177605 | 0.09 | 0.09 | 0.04 | 94.37 | 86.96 | 59.85 |
| CO2_1 | FKDN220186440-1A | 158864 | 155889 | 0.08 | 0.08 | 0.03 | 95.71 | 89.53 | 55.59 |
| Flood_1 | FKDN220186449-1A | 179382 | 149829 | 0.09 | 0.07 | 0.03 | 96.97 | 92.27 | 57.88 |
| CO2_3 | FKDN220186442-1A | 157217 | 150948 | 0.08 | 0.08 | 0.03 | 97.09 | 92.53 | 47.25 |
| Control_2 | FKDN220186459-1A | 169288 | 169061 | 0.08 | 0.08 | 0.03 | 96.29 | 90.88 | 52.31 |

**Table S5**: Novogene filtering and quality control F003, ITS shoot and root samples.

| **Sample** | **NovoID** | **Raw Reads** | **Clean Reads** | **Raw Base (G)** | **Clean Base (G)** | **Error Rate (%)** | **Q20 (%)** | **Q30 (%)** | **GC Content (%)** |
| --- | --- | --- | --- | --- | --- | --- | --- | --- | --- |
| CO2_L3 | FKDN220186439-1A | 168599 | 167732 | 0.08 | 0.08 | 0.03 | 97.15 | 92.24 | 50.65 |
| R1C | FKDN220186452-1A | 179108 | 178979 | 0.09 | 0.09 | 0.03 | 97.19 | 92.51 | 47.01 |
| R2C | FKDN220186453-1A | 166885 | 166816 | 0.08 | 0.08 | 0.03 | 97.25 | 92.56 | 46.82 |
| FL3 | FKDN220186448-1A | 167159 | 151060 | 0.08 | 0.08 | 0.03 | 96.96 | 91.18 | 58.7 |
| FR1 | FKDN220186443-1A | 164490 | 164371 | 0.08 | 0.08 | 0.03 | 97.07 | 91.94 | 48.16 |
| FR2 | FKDN220186444-1A | 180968 | 180903 | 0.09 | 0.09 | 0.03 | 97.03 | 91.85 | 48.2 |
| L3C | FKDN220186457-1A | 188447 | 187655 | 0.09 | 0.09 | 0.03 | 97.22 | 92.27 | 49.94 |
| FCO2_R1 | FKDN220186425-1A | 163025 | 162949 | 0.08 | 0.08 | 0.03 | 97.41 | 92.81 | 52.34 |
| FCO2_R2 | FKDN220186426-1A | 179100 | 179066 | 0.09 | 0.09 | 0.03 | 97.41 | 92.81 | 52.19 |
| FCO2_R3 | FKDN220186427-1A | 176455 | 175949 | 0.09 | 0.09 | 0.03 | 97.31 | 92.62 | 52.28 |
| FCO2_L1 | FKDN220186428-1A | 181870 | 181472 | 0.09 | 0.09 | 0.03 | 97.45 | 92.85 | 50.08 |
| FCO2_L2 | FKDN220186429-1A | 184802 | 184387 | 0.09 | 0.09 | 0.03 | 97.45 | 92.82 | 49.05 |
| FCO2_L3 | FKDN220186430-1A | 186210 | 185717 | 0.09 | 0.09 | 0.03 | 97.3 | 92.57 | 50.92 |
| L2C | FKDN220186456-1A | 170920 | 170166 | 0.09 | 0.09 | 0.03 | 97.01 | 91.75 | 49.7 |
| L1C | FKDN220186455-1A | 166747 | 165495 | 0.08 | 0.08 | 0.03 | 97.15 | 92.06 | 51.02 |
| R3C | FKDN220186454-1A | 162493 | 161885 | 0.08 | 0.08 | 0.03 | 97.21 | 92.48 | 46.42 |
| CO2_R1 | FKDN220186434-1A | 161748 | 161730 | 0.08 | 0.08 | 0.03 | 97.5 | 93.18 | 43.88 |
| CO2_R2 | FKDN220186435-1A | 167556 | 167528 | 0.08 | 0.08 | 0.03 | 97.44 | 93.1 | 44.64 |
| CO2_R3 | FKDN220186436-1A | 140959 | 140514 | 0.07 | 0.07 | 0.03 | 97.45 | 93.06 | 45.07 |
| CO2_L1 | FKDN220186437-1A | 159981 | 159477 | 0.08 | 0.08 | 0.03 | 97.03 | 92.1 | 50.5 |
| CO2_L2 | FKDN220186438-1A | 171671 | 169057 | 0.09 | 0.08 | 0.03 | 97.26 | 92.44 | 51.19 |
| FR3 | FKDN220186445-1A | 179699 | 179655 | 0.09 | 0.09 | 0.03 | 96.95 | 91.68 | 48.01 |
| FL1 | FKDN220186446-1A | 171601 | 168058 | 0.09 | 0.08 | 0.03 | 96.95 | 91.73 | 48.53 |
| FL2 | FKDN220186447-1A | 151870 | 150330 | 0.08 | 0.08 | 0.03 | 96.56 | 91.12 | 50.74 |

**Table S6**: denoising, merging and chimera check for F001, 16S soil samples.

| **sample-id** | **input** | **filtered** | **percentage of input passed filter** | **denoised** | **merged** | **percentage of input merged** | **non-chimeric** | **percentage of input non-chimeric** |
| --- | --- | --- | --- | --- | --- | --- | --- | --- |
| CO2_1 | 177887 | 163902 | 92.14 | 162343 | 155589 | 87.47 | 150651 | 84.69 |
| CO2_2 | 178807 | 166203 | 92.95 | 163425 | 146078 | 81.7 | 116745 | 65.29 |
| CO2_3 | 180298 | 166400 | 92.29 | 164406 | 154404 | 85.64 | 148278 | 82.24 |
| Control_1 | 161226 | 147396 | 91.42 | 146217 | 140307 | 87.03 | 135416 | 83.99 |
| Control_2 | 183047 | 168150 | 91.86 | 166175 | 155595 | 85 | 131890 | 72.05 |
| Control_3 | 163795 | 150323 | 91.78 | 148703 | 139033 | 84.88 | 131294 | 80.16 |
| F_C_1 | 169697 | 155606 | 91.7 | 154534 | 150117 | 88.46 | 146085 | 86.09 |
| F_C_2 | 186485 | 172451 | 92.47 | 171108 | 165838 | 88.93 | 159574 | 85.57 |
| F_C_3 | 169962 | 156662 | 92.17 | 154857 | 146550 | 86.23 | 140998 | 82.96 |
| Flood_1 | 174799 | 160879 | 92.04 | 159044 | 151596 | 86.73 | 144635 | 82.74 |
| Flood_2 | 174015 | 159915 | 91.9 | 158032 | 150094 | 86.25 | 142080 | 81.65 |
| Flood_3 | 176631 | 162728 | 92.13 | 161012 | 153262 | 86.77 | 147350 | 83.42 |

**Table S7**: denoising, merging and chimera check for F002, 16S shoot and root samples.

| **sample-id** | **input** | **filtered** | **percentage of input passed filter** | **denoised** | **merged** | **percentage of input merged** | **non-chimeric** | **percentage of input non-chimeric** |
| --- | --- | --- | --- | --- | --- | --- | --- | --- |
| CO2_L1 | 167928 | 156011 | 92.9 | 155230 | 153292 | 91.28 | 150347 | 89.53 |
| CO2_L2 | 151021 | 140543 | 93.06 | 139833 | 137755 | 91.22 | 134443 | 89.02 |
| CO2_L3 | 166459 | 154714 | 92.94 | 153840 | 151357 | 90.93 | 145877 | 87.64 |
| CO2_R1 | 182119 | 167154 | 91.78 | 160825 | 136069 | 74.71 | 70519 | 38.72 |
| CO2_R2 | 181668 | 166670 | 91.74 | 163427 | 150577 | 82.89 | 134982 | 74.3 |
| CO2_R3 | 183575 | 167102 | 91.03 | 163298 | 150084 | 81.76 | 130364 | 71.01 |
| FC_L1 | 161978 | 148877 | 91.91 | 148132 | 141899 | 87.6 | 128282 | 79.2 |
| FC_L2 | 183482 | 168679 | 91.93 | 167952 | 162141 | 88.37 | 151063 | 82.33 |
| FC_L3 | 171701 | 157468 | 91.71 | 156864 | 148586 | 86.54 | 133214 | 77.58 |
| FC_R1 | 170609 | 153619 | 90.04 | 150814 | 136421 | 79.96 | 117339 | 68.78 |
| FC_R2 | 171035 | 154800 | 90.51 | 152086 | 138237 | 80.82 | 120571 | 70.49 |
| FC_R3 | 179599 | 163788 | 91.2 | 161675 | 151469 | 84.34 | 139537 | 77.69 |
| FL1 | 158426 | 147655 | 93.2 | 146956 | 145326 | 91.73 | 138649 | 87.52 |
| FL2 | 189531 | 176358 | 93.05 | 175747 | 174370 | 92 | 169173 | 89.26 |
| FL3 | 162967 | 151382 | 92.89 | 151079 | 150706 | 92.48 | 146932 | 90.16 |
| FR1 | 169295 | 155123 | 91.63 | 149795 | 120412 | 71.13 | 60570 | 35.78 |
| FR2 | 187806 | 172118 | 91.65 | 166757 | 136863 | 72.87 | 65606 | 34.93 |
| FR3 | 186798 | 169155 | 90.56 | 165720 | 147799 | 79.12 | 107487 | 57.54 |
| L1C | 152392 | 141634 | 92.94 | 140844 | 138871 | 91.13 | 135394 | 88.85 |
| L2C | 182280 | 169697 | 93.1 | 168881 | 166851 | 91.54 | 163401 | 89.64 |
| L3C | 160649 | 149103 | 92.81 | 148358 | 146184 | 91 | 141053 | 87.8 |
| R1C | 170404 | 153802 | 90.26 | 149781 | 135188 | 79.33 | 117906 | 69.19 |
| R2C | 165551 | 151032 | 91.23 | 147682 | 136652 | 82.54 | 123423 | 74.55 |
| R3C | 171072 | 156826 | 91.67 | 154035 | 144857 | 84.68 | 135389 | 79.14 |

**Table S8**: denoising, merging and chimera check for F005, ITS soil samples.

| **sample-id** | **input** | **filtered** | **percentage of input passed filter** | **denoised** | **merged** | **percentage of input merged** | **non-chimeric** | **percentage of input non-chimeric** |
| --- | --- | --- | --- | --- | --- | --- | --- | --- |
| CO2_1 | 158864 | 131219 | 82.6 | 128693 | 125630 | 79.08 | 121374 | 76.4 |
| CO2_3 | 157217 | 136943 | 87.1 | 135078 | 128164 | 81.52 | 126330 | 80.35 |
| Control_1 | 188754 | 162069 | 85.86 | 161243 | 157718 | 83.56 | 154076 | 81.63 |
| Control_2 | 169288 | 143856 | 84.98 | 143172 | 140007 | 82.7 | 132634 | 78.35 |
| F_C_1 | 175692 | 141583 | 80.59 | 139504 | 134487 | 76.55 | 127155 | 72.37 |
| F_C_2 | 186689 | 136772 | 73.26 | 132239 | 125704 | 67.33 | 120287 | 64.43 |
| F_C_3 | 169656 | 135482 | 79.86 | 133988 | 130304 | 76.8 | 125591 | 74.03 |
| Flood_1 | 179382 | 144910 | 80.78 | 140573 | 127345 | 70.99 | 113396 | 63.21 |
| Flood_2 | 161623 | 133860 | 82.82 | 129436 | 123383 | 76.34 | 106485 | 65.88 |
| Flood_3 | 188670 | 139273 | 73.82 | 135333 | 129248 | 68.5 | 105500 | 55.92 |

**Table S9:** denoising, merging and chimera check for F003, ITS shoot and root samples.

| **sample-id** | **input** | **filtered** | **percentage of input passed filter** | **denoised** | **merged** | **percentage of input merged** | **non-chimeric** | **percentage of input non-chimeric** |
| --- | --- | --- | --- | --- | --- | --- | --- | --- |
| CO2_L1 | 159981 | 142809 | 89.27 | 142510 | 141698 | 88.57 | 140871 | 88.05 |
| CO2_L2 | 171671 | 155038 | 90.31 | 154150 | 152668 | 88.93 | 151880 | 88.47 |
| CO2_L3 | 168599 | 152536 | 90.47 | 151967 | 150889 | 89.5 | 150196 | 89.08 |
| CO2_R1 | 161748 | 145176 | 89.75 | 144953 | 144547 | 89.37 | 144475 | 89.32 |
| CO2_R2 | 167556 | 150102 | 89.58 | 149921 | 149249 | 89.07 | 148857 | 88.84 |
| CO2_R3 | 140959 | 126660 | 89.86 | 126214 | 125225 | 88.84 | 123712 | 87.76 |
| FCO2_L1 | 181870 | 166329 | 91.45 | 165656 | 164634 | 90.52 | 163007 | 89.63 |
| FCO2_L2 | 184802 | 168454 | 91.15 | 167831 | 166405 | 90.05 | 164286 | 88.9 |
| FCO2_L3 | 186210 | 169205 | 90.87 | 168481 | 167378 | 89.89 | 165655 | 88.96 |
| FCO2_R1 | 163025 | 150204 | 92.14 | 150027 | 149648 | 91.79 | 149292 | 91.58 |
| FCO2_R2 | 179100 | 164819 | 92.03 | 164602 | 164151 | 91.65 | 163733 | 91.42 |
| FCO2_R3 | 176455 | 161738 | 91.66 | 161289 | 160470 | 90.94 | 159839 | 90.58 |
| FL1 | 171601 | 153680 | 89.56 | 152793 | 151333 | 88.19 | 150068 | 87.45 |
| FL2 | 151870 | 133610 | 87.98 | 132693 | 131039 | 86.28 | 129741 | 85.43 |
| FL3 | 167159 | 146336 | 87.54 | 144780 | 141131 | 84.43 | 139303 | 83.34 |
| FR1 | 164490 | 150296 | 91.37 | 149949 | 149057 | 90.62 | 146744 | 89.21 |
| FR2 | 180968 | 164933 | 91.14 | 164701 | 163523 | 90.36 | 161599 | 89.3 |
| FR3 | 179699 | 163770 | 91.14 | 163655 | 162585 | 90.48 | 161739 | 90.01 |
| L1C | 166747 | 150823 | 90.45 | 150119 | 148501 | 89.06 | 148138 | 88.84 |
| L2C | 170920 | 155006 | 90.69 | 154403 | 153356 | 89.72 | 149976 | 87.75 |
| L3C | 188447 | 170659 | 90.56 | 169946 | 168467 | 89.4 | 168098 | 89.2 |
| R1C | 179108 | 160247 | 89.47 | 160060 | 159447 | 89.02 | 159252 | 88.91 |
| R2C | 166885 | 150429 | 90.14 | 150231 | 149449 | 89.55 | 149211 | 89.41 |
| R3C | 162493 | 145756 | 89.7 | 145218 | 143739 | 88.46 | 143246 | 88.16 |

**Table S10:** ASV counts.

|  | | Control | CO2 | Flooding + CO2 | Flooding |
| --- | --- | --- | --- | --- | --- |
| Bacterial (16S) | Soil | 407,775 | 395,286 | 445,424 | 433,288 |
|  | Root | 345,594 | 380,432 | 421,705 | 309,938 |
|  | Shoot | 436,886 | 448,289 | 434,248 | 463,021 |
|  | Total | 1,190,255 | 1,224,007 | 1,301,377 | 1,206,247 |
| Fungal (ITS) | Soil | 198,732 | 230,816 | 344,389 | 116,326 |
|  | Root | 336,065 | 418,822 | 413,417 | 460,892 |
|  | Shoot | 409,182 | 411,483 | 488,375 | 393,257 |
|  | Total | 943,979 | 1,061,121 | 1,246,181 | 970,475 |

**Table S11:** Shannon vector values

| **Sample ID** | | | **Shannon entropy** | **Average** |
| --- | --- | --- | --- | --- |
| **Fungi** | **Shoot** | **Control-1** | **2.793720564** | **2.8889061** |
|  |  | **Control-2** | **2.847367437** |  |
|  |  | **Control-3** | **3.025630186** |  |
|  |  | **CO2 -1** | **2.840982608** | **2.8027604** |
|  |  | **CO2 -2** | **2.729409269** |  |
|  |  | **CO2 -3** | **2.837889214** |  |
|  |  | **Flooding + CO2 -1** | **2.528235168** | **2.553284** |
|  |  | **Flooding + CO2 -2** | **2.450388453** |  |
|  |  | **Flooding + CO2 -3** | **2.681228315** |  |
|  |  | **Flooding -1** | **4.685432917** | **3.6631749** |
|  |  | **Flooding -2** | **4.732318859** |  |
|  |  | **Flooding -3** | **1.571772983** |  |
|  | **Root** | **Control -1** | **2.779060895** | **2.7961939** |
|  |  | **Control -2** | **2.750811947** |  |
|  |  | **Control -3** | **2.858708948** |  |
|  |  | **CO2 -1** | **1.80186702** | **1.9479611** |
|  |  | **CO2 -2** | **1.931182686** |  |
|  |  | **CO2 -3** | **2.110833446** |  |
|  |  | **Flooding + CO2 -1** | **0.753117507** | **0.6797407** |
|  |  | **Flooding + CO2 -2** | **0.328869575** |  |
|  |  | **Flooding + CO2 -3** | **0.95723497** |  |
|  |  | **Flooding -1** | **2.129216176** | **2.197736** |
|  |  | **Flooding -2** | **2.193015429** |  |
|  |  | **Flooding -3** | **2.270976305** |  |
|  | **Soil** | **Control -1** | **3.451457019** | **3.6466776** |
|  |  | **Control -2** | **3.841898246** |  |
|  |  | **Control -3** |  |  |
|  |  | **CO2 -1** | **3.934720195** | **3.5781547** |
|  |  | **CO2 -2** |  |  |
|  |  | **CO2 -3** | **3.221589139** |  |
|  |  | **Flooding + CO2 -1** | **3.498703975** | **3.6679879** |
|  |  | **Flooding + CO2 -2** | **3.461713881** |  |
|  |  | **Flooding + CO2 -3** | **4.043545774** |  |
|  |  | **Flooding -1** | **3.733514984** | **3.6700592** |
|  |  | **Flooding -2** | **3.588017871** |  |
|  |  | **Flooding -3** | **3.6886448** |  |
| **Bacteria** | **Shoot** | **Control-1** | **0.719329586** | **0.70132** |
|  |  | **Control-2** | **0.62538432** |  |
|  |  | **Control-3** | **0.759245984** |  |
|  |  | **CO2 -1** | **0.737115268** | **0.7849923** |
|  |  | **CO2 -2** | **0.813515816** |  |
|  |  | **CO2 -3** | **0.804345783** |  |
|  |  | **Flooding + CO2 -1** | **1.904471767** | **1.6969729** |
|  |  | **Flooding + CO2 -2** | **1.729815532** |  |
|  |  | **Flooding + CO2 -3** | **1.456631501** |  |
|  |  | **Flooding -1** | **0.739524944** | **0.5244001** |
|  |  | **Flooding -2** | **0.491874841** |  |
|  |  | **Flooding -3** | **0.341800554** |  |
|  | **Root** | **Control -1** | **6.575702203** | **5.6822698** |
|  |  | **Control -2** | **5.328634619** |  |
|  |  | **Control -3** | **5.142472604** |  |
|  |  | **CO2 -1** | **6.043346992** | **6.1818344** |
|  |  | **CO2 -2** | **6.412474769** |  |
|  |  | **CO2 -3** | **6.089681574** |  |
|  |  | **Flooding + CO2 -1** | **6.383921653** | **6.0352542** |
|  |  | **Flooding + CO2 -2** | **6.092337271** |  |
|  |  | **Flooding + CO2 -3** | **5.629503578** |  |
|  |  | **Flooding -1** | **5.859715029** | **5.6672238** |
|  |  | **Flooding -2** | **5.563559078** |  |
|  |  | **Flooding -3** | **5.578397426** |  |
|  | **Soil** | **Control -1** | **3.666322642** | **4.2596094** |
|  |  | **Control -2** | **1.410432931** |  |
|  |  | **Control -3** | **7.702072495** |  |
|  |  | **CO2 -1** | **5.518197857** | **6.0811961** |
|  |  | **CO2 -2** | **5.953367954** |  |
|  |  | **CO2 -3** | **6.772022418** |  |
|  |  | **Flooding + CO2 -1** | **4.037746509** | **5.1600275** |
|  |  | **Flooding + CO2 -2** | **4.317601038** |  |
|  |  | **Flooding + CO2 -3** | **7.124735033** |  |
|  |  | **Flooding -1** | **5.689995647** | **5.7526192** |
|  |  | **Flooding -2** | **5.752106105** |  |
|  |  | **Flooding -3** | **5.81575593** |  |
